# Integrated CT Foundation Model and Radiomic Features Define Clinically and Biologically Distinct Colorectal Cancer Subtypes

**DOI:** 10.34133/research.1435

**Published:** 2026-10-06

**Authors:** Du Cai, Haoning Qi, Qiuxia Yang, Yibin Zhao, Chenghang Li, Chuling Hu, Yinmeng Zhang, Rende Huang, Wenjun Mao, Xiaojian Wu, Yize Mao, Feng Gao

**Affiliations:** ^1^ Department of General Surgery (Colorectal Surgery), The Sixth Affiliated Hospital, Sun Yat-sen University, Guangzhou, P. R. China.; ^2^ Guangdong Provincial Key Laboratory of Colorectal and Pelvic Floor Diseases, The Sixth Affiliated Hospital, Sun Yat-sen University, Guangzhou, P. R. China.; ^3^ Biomedical Innovation Center, The Sixth Affiliated Hospital, Sun Yat-sen University, Guangzhou, P. R. China.; ^4^ Department of Radiology, State Key Laboratory of Oncology in South China, Guangdong Provincial Clinical Research Center for Cancer, Sun Yat-sen University Cancer Center, Guangzhou 510060, P. R. China.; ^5^ Department of Colorectal Surgery, Ningbo Medical Center Lihuili Hospital (Affiliated Lihuili Hospital of Ningbo University), Ningbo 315000, P. R. China.; ^6^ Artificial Intelligence Thrust, The Hong Kong University of Science and Technology, Guangzhou, P. R. China.; ^7^ Department of Gastroenterology, The First Hospital of Jilin University, Changchun, P. R. China.; ^8^ Department of Thoracic Surgery, The Affiliated Wuxi People’s Hospital of Nanjing Medical University, Wuxi People’s Hospital, Wuxi Medical Center, Nanjing Medical University, Wuxi 214023, P. R. China.; ^9^ Department of Pancreatobiliary Surgery, State Key Laboratory of Oncology in South China, Guangdong Provincial Clinical Research Center for Cancer, Sun Yat-sen University Cancer Center, Guangzhou 510060, P. R. China.

## Abstract

Colorectal cancer is clinically heterogeneous, and current risk stratification does not fully capture differences in recurrence, survival, or treatment response. Molecular subtyping has improved biological classification, but sequencing-based workflows are not always available before treatment or at the population scale. We developed a survival-informed computed tomography (CT) phenotyping framework that combines CT foundation-model representations with PyRadiomics descriptors of tumor shape, intensity, and texture. Across 4 nonoverlapping cohorts, including SYSU6H cohort 1 (*N* = 902), SYSU6H cohort 2 (*N* = 3,267), external Ningbo cohort 1 (*N* = 509), and SYSU6HNAT neoadjuvant-treatment cohort (*N* = 709), fused CT features were selected and integrated to derive survival-informed CT imaging phenotypes, followed by evaluation of survival, adjusted prognostic associations, molecular correlates, phenotype assignment, and treatment response. The fused representation identified 2 survival-informed imaging phenotypes with marked survival differences. Compared with CT-S1, CT-S2 was associated with worse disease-free survival and overall survival (disease-free survival hazard ratio = 11.16, 95% confidence interval 9.19 to 13.55; overall survival hazard ratio = 14.29, 95% confidence interval 10.83 to 18.85; both *P* < 0.0001) and remained associated with outcomes after adjustment for clinicopathologic variables and measured tumor size. CT-S2 was enriched for consensus molecular subtype 4-like composition and stromal/extracellular matrix programs, suggesting transcriptomic and microenvironment-associated correlates linked to the adverse imaging phenotype. A flexible phenotype-assignment model reproduced prognostic separation in internal and external cohorts, and assigned phenotypes were associated with survival and pathological response in SYSU6HNAT. These findings suggest that integrated CT phenotyping may provide a noninvasive approach for identifying survival-informed colorectal cancer imaging phenotypes with exploratory molecular correlates, supporting further independent validation.

## Introduction

Colorectal cancer (CRC) remains a major clinical problem because patients with similar anatomical stage can experience different risks of recurrence, survival, and treatment response [1–3]. Molecular subtyping has helped organize this heterogeneity into biologically meaningful programs, including epithelial, immune, metabolic, mesenchymal, and stromal states [4–6]. However, molecular classification requires tumor tissue, sequencing infrastructure, and adequate sample quality, which can limit its routine use before treatment decisions or at the population scale. These constraints create a need for noninvasive imaging phenotypes that capture clinically relevant tumor heterogeneity from data already available in routine care.

Routine computed tomography (CT) imaging offers a practical route to such phenotyping. Radiomics studies have shown that quantitative CT features can stratify tumors into image-derived subtypes associated with prognosis and biological state, including in unsupervised clustering settings [7–11]. Most existing imaging subtype studies, however, rely on handcrafted radiomic descriptors. These features are interpretable but predefined, compressing complex images into a finite set of human-designed measurements and limiting the ability to capture latent patterns learned from large-scale imaging data.

Self-supervised foundation models can address part of this limitation by learning high-capacity image representations from large datasets and improving performance across clinical prediction tasks [12,13]. For imaging phenotype derivation, however, deep representations introduce a different challenge: They may separate patients effectively but remain difficult to interpret or relate to familiar imaging and biological concepts. An integrated CT representation that combines handcrafted radiomic descriptors with foundation-model features may therefore expand feature capacity while retaining an interpretable basis for biological and clinical characterization [7–13].

Here, we developed an integrated CT phenotyping framework for CRC by combining radiomic features and foundation-model representations extracted from the same tumor region. We used this framework to derive survival-informed CT imaging phenotypes, tested their prognostic value beyond established clinicopathologic variables, linked them to consensus molecular subtype (CMS) composition and transcriptomic programs, and evaluated phenotype assignment in external and neoadjuvant-treatment cohorts. This study asks whether routine CT images can support a scalable imaging-based phenotyping framework for clinically relevant CRC risk stratification while connecting imaging phenotypes to clinical, molecular, and treatment-response contexts.

## Results

### Study cohorts and an integrated CT phenotyping framework

We designed a multicohort study to convert routinely acquired venous-phase CT scans into a noninvasive CRC imaging-phenotype framework and to evaluate the framework across phenotype derivation, prognosis, biological interpretation, and clinical application (Fig. 1). The 4 nonoverlapping cohorts were analyzed according to predefined project roles and data availability rather than a single chronological split. SYSU6H cohort 1 was a CT-only cohort used for CRCFound fine-tuning (*N* = 902). SYSU6H cohort 2 was an independent multimodal cohort with linked tissue and omics resources and served as the primary cohort for survival-informed imaging phenotype derivation and survival analysis (*N* = 3,267). Ningbo cohort 1 served as an institutionally independent external evaluation cohort (*N* = 509), whereas SYSU6HNAT was used for evaluation of treatment response after neoadjuvant therapy (*N* = 709). Survival analyses were based on disease-free survival (DFS) and overall survival (OS), and the neoadjuvant cohort was additionally evaluated using pathological complete response (pCR) and tumor regression grade (TRG).

**Fig. 1.**
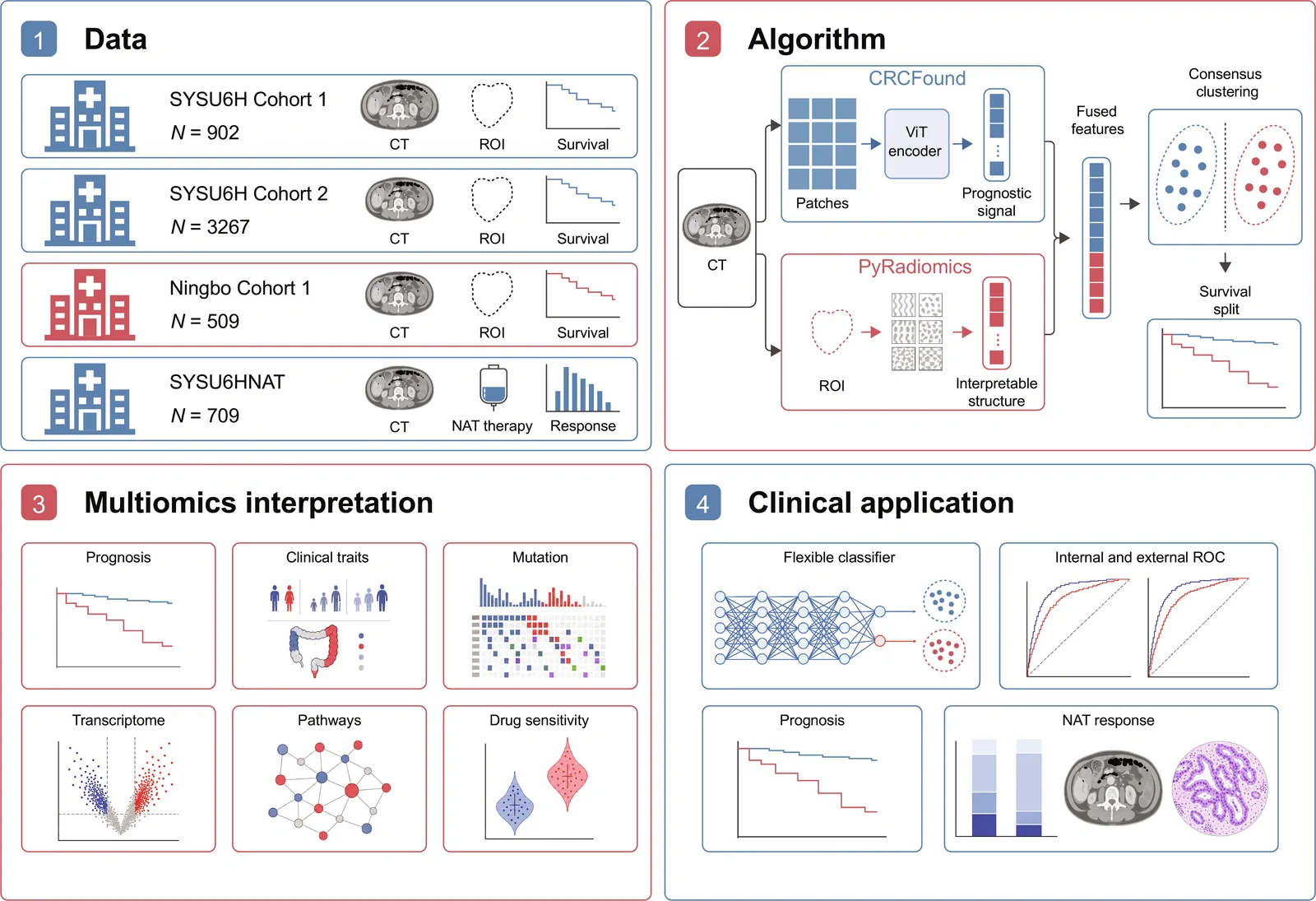
Study overview and integrated computed tomography (CT) phenotyping framework. Schematic overview of the multicohort integrated CT phenotyping framework. SYSU6H cohort 1 (*N* = 902) was used for CRCFound survival fine-tuning and representation adaptation. SYSU6H cohort 2 (*N* = 3,267) was used for feature fusion, survival-informed imaging phenotype derivation, primary prognostic evaluation, clinicopathologic association, and biological interpretation. Ningbo cohort 1 (*N* = 509) served as an external evaluation cohort. SYSU6HNAT (*N* = 709) was used to assess the relevance of assigned CT phenotypes in the neoadjuvant-treatment setting, including disease-free survival (DFS), overall survival (OS), pathological complete response (pCR), and tumor regression grade (TRG). CRCFound extracts deep foundation-model representations, whereas PyRadiomics extracts interpretable handcrafted descriptors; fused features are used to derive survival-informed CT imaging phenotypes and evaluate prognosis, molecular correlates, and clinical application. NAT, neoadjuvant treatment.

Table S1 summarizes the cohort design and baseline clinical characteristics. Because SYSU6H cohort 1 was used exclusively for CRCFound representation adaptation and did not receive CT-phenotype assignments, its baseline characteristics are reported for the cohort overall. For cohorts with phenotype assignments, baseline characteristics are additionally stratified by CT phenotype, including age, body mass index (BMI) where available, follow-up time, sex, tumor side, grade, TNM or clinical TNM stage, microsatellite instability/mismatch repair (MSI/MMR) status, and DFS/OS event status. Table S2 further summarizes cohort roles and center-specific CT scanner and acquisition characteristics. Across the phenotype-analyzed cohorts, CT-S2 generally showed a less favorable clinical profile, with higher survival event rates and, in SYSU6H cohort 2, a larger proportion of stage IV tumors (16.0% versus 2.6% in CT-S1).

The framework combined 2 complementary CT representations from the same tumor region of interest (ROI): CRCFound-derived deep features [13] and PyRadiomics-derived handcrafted descriptors [10,11]. The 2 feature sources were first evaluated separately and then fused to derive survival-informed imaging phenotypes, which were subsequently assessed across survival, clinicopathologic, molecular, and treatment-response analyses.

In a random 30-case subset of SYSU6H cohort 2, the mean interobserver Dice similarity coefficient (DSC) for segmentation reproducibility was 0.924 ± 0.053. The intraclass correlation coefficient [ICC (2,1)] was 0.977 (95% confidence interval [CI] 0.951 to 0.990) for tumor volume and 0.985 (95% CI 0.953 to 0.999) for maximum axial diameter. Across 1,326 nonconstant PyRadiomics features with valid estimates, median ICC was 0.972 (interquartile range [IQR], 0.943 to 0.987); all features had ICC ≥ 0.75 and 92.8% had ICC ≥ 0.90 (Fig. S1 and Table S3).

### Integrated CT features identify 2 survival-informed CRC imaging phenotypes

We first compared CRCFound-only, PyRadiomics-only, and fused CRCFound-PyRadiomics representations for imaging-phenotype derivation using consensus clustering (Fig. 2A). The single-branch analyses showed complementary behavior. CRCFound-only clustering produced stronger survival separation but weaker geometric separation in the feature space (silhouette = 0.255; DFS hazard ratio [HR] = 6.73, 95% CI 5.70 to 7.95; OS HR = 7.66, 95% CI 6.15 to 9.54). PyRadiomics-only clustering produced more compact feature-space separation (silhouette = 0.422) but weaker survival separation (DFS HR = 1.39, 95% CI 1.21 to 1.61; OS HR = 1.78, 95% CI 1.47 to 2.16).

**Fig. 2.**
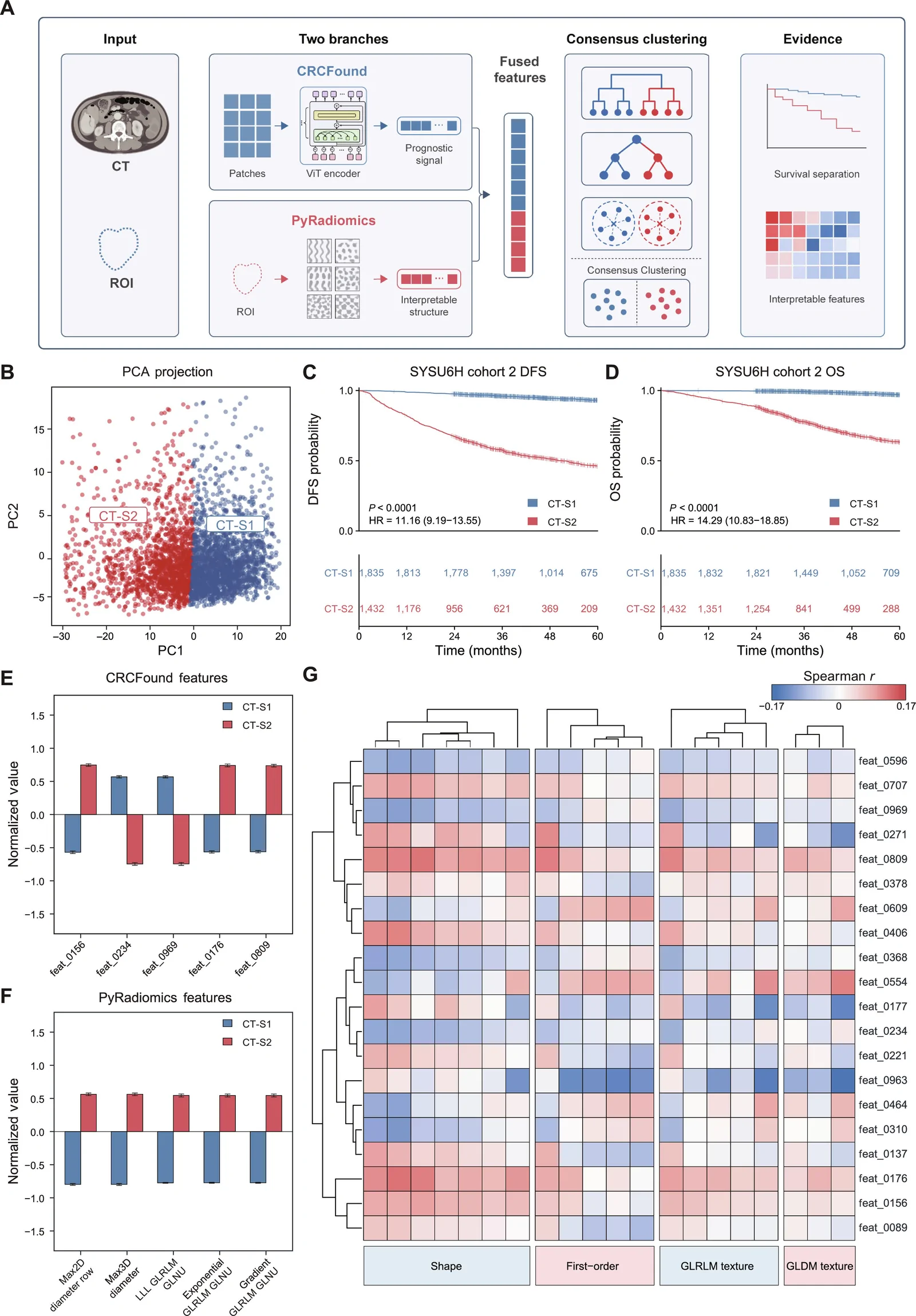
Integrated CRCFound and PyRadiomics features identify survival-informed computed tomography (CT) imaging phenotypes. (A) Workflow for dual-branch feature extraction, fusion, consensus clustering, phenotype identification, and downstream evidence evaluation. (B) Principal component analysis (PCA) projection of fused features in the SYSU6H cohort 2, colored by CT phenotype. (C and D) Kaplan–Meier curves for disease-free survival (DFS) (C) and overall survival (OS) (D) by fused CT phenotype in SYSU6H cohort 2. (E and F) Normalized distributions of top phenotype-associated CRCFound (E) and PyRadiomics (F) features. (G) Cross-modality Spearman correlation heatmap between selected PyRadiomics and CRCFound features, with PyRadiomics features grouped by radiomic category. Hazard ratios are from Cox models; *P* values are from log-rank tests unless otherwise specified.

The fused representation identified 2 major survival-informed CT imaging phenotypes in SYSU6H cohort 2, referred to as CT-S1 and CT-S2. Survival information was incorporated during CRCFound representation adaptation and feature screening, whereas consensus clustering was performed using the selected imaging features alone. Principal component visualization showed separation between the 2 groups (Fig. 2B). CT-S2 was associated with substantially worse DFS and OS than CT-S1 (DFS HR = 11.16, 95% CI 9.19 to 13.55; OS HR = 14.29, 95% CI 10.83 to 18.85; both *P* < 0.0001; Fig. 2C and D). Analyses across *k* = 2 to 8 supported *k* = 2 as a parsimonious solution within this survival-informed workflow (Fig. S2). These findings define 2 clinically interpretable, survival-informed imaging phenotypes that can be further characterized through their clinical and exploratory biological correlates.

Phenotype-associated feature inspection showed that both CRCFound and PyRadiomics features differed between CT-S1 and CT-S2 (Fig. 2E and F). Correlation analysis between representative CRCFound features and radiomic descriptors suggested that the 2 branches were not simple duplicates of each other (Fig. 2G). This supports the use of feature fusion as a representation strategy, although the biological meaning of individual deep features should be interpreted cautiously.

To examine dependence on outcome-guided screening, we repeated feature processing and consensus clustering without the modality-specific univariable Cox feature-screening step. In the same SYSU6H cohort 2, the DFS and OS C-indices were 0.756 and 0.769 with Cox feature screening, compared with 0.501 and 0.506 without screening, respectively. The sensitivity solution also showed fewer Hallmark pathway differences (2 of 50 versus 11 of 50 pathways at a Benjamini–Hochberg false discovery rate (BH-FDR) < 0.05; Fig. S3A to C and Table S4), confirming that the principal prognostic separation depended on the survival-informed feature-selection framework.

### Survival-informed CT phenotypes add prognostic information beyond clinicopathologic factors

We next examined whether the survival-informed CT phenotypes provided prognostic information beyond established clinical variables and tumor size. In multivariable Cox models including TNM stage, MSI status, tumor side, grade, BMI, sex, age, ROI-derived maximum axial diameter, and tumor volume, CT-S2 remained associated with worse DFS (HR = 8.80, 95% CI 6.93 to 11.19) and OS (HR = 10.91, 95% CI 7.46 to 15.96; both *P* < 0.001; Fig. 3A and B). CT-S2 also showed larger tumor volume and maximum axial diameter in SYSU6H cohort 2 (both *P* < 0.001) and Ningbo cohort 1 (*P* = 0.006 and *P* = 0.011, respectively; Fig. S4A and B and Table S5). In an unpenalized complete-case multivariable Cox model in Ningbo cohort 1 that included CT phenotype, age, sex, grouped TNM stage, maximum axial diameter, and tumor volume, CT-S2 remained associated with worse DFS (HR = 13.92, 95% CI 6.01 to 32.20) and OS (HR = 20.24, 95% CI 4.86 to 84.26; both *P* < 0.001; Table S5). In paired analyses using the same complete-case sets and covariate definitions as the principal SYSU6H models, adding tumor-size covariates produced only small changes in the CT-S2 HRs in SYSU6H cohort 2 (DFS, 8.85 to 8.80; OS, 11.32 to 10.91; Table S5) and Ningbo cohort 1 (DFS, 15.04 to 13.92; OS, 21.09 to 20.24; Table S5). Figure 3A and B shows the principal models including tumor-size covariates, whereas Fig. S4C and D shows the corresponding models without tumor-size covariates; the estimates differed little. These findings indicate that the prognostic association of the CT phenotype persisted after adjustment for measured tumor size. Contribution analysis further suggested that the phenotype adds a major risk axis to clinical models. In models without the CT phenotype, TNM stage accounted for most of the model contribution for DFS and OS. After CT phenotype was included, phenotype became a major contributor while TNM stage remained important (Fig. 3C to F). Together, CT phenotype and TNM stage contributed complementary prognostic information.

**Fig. 3.**
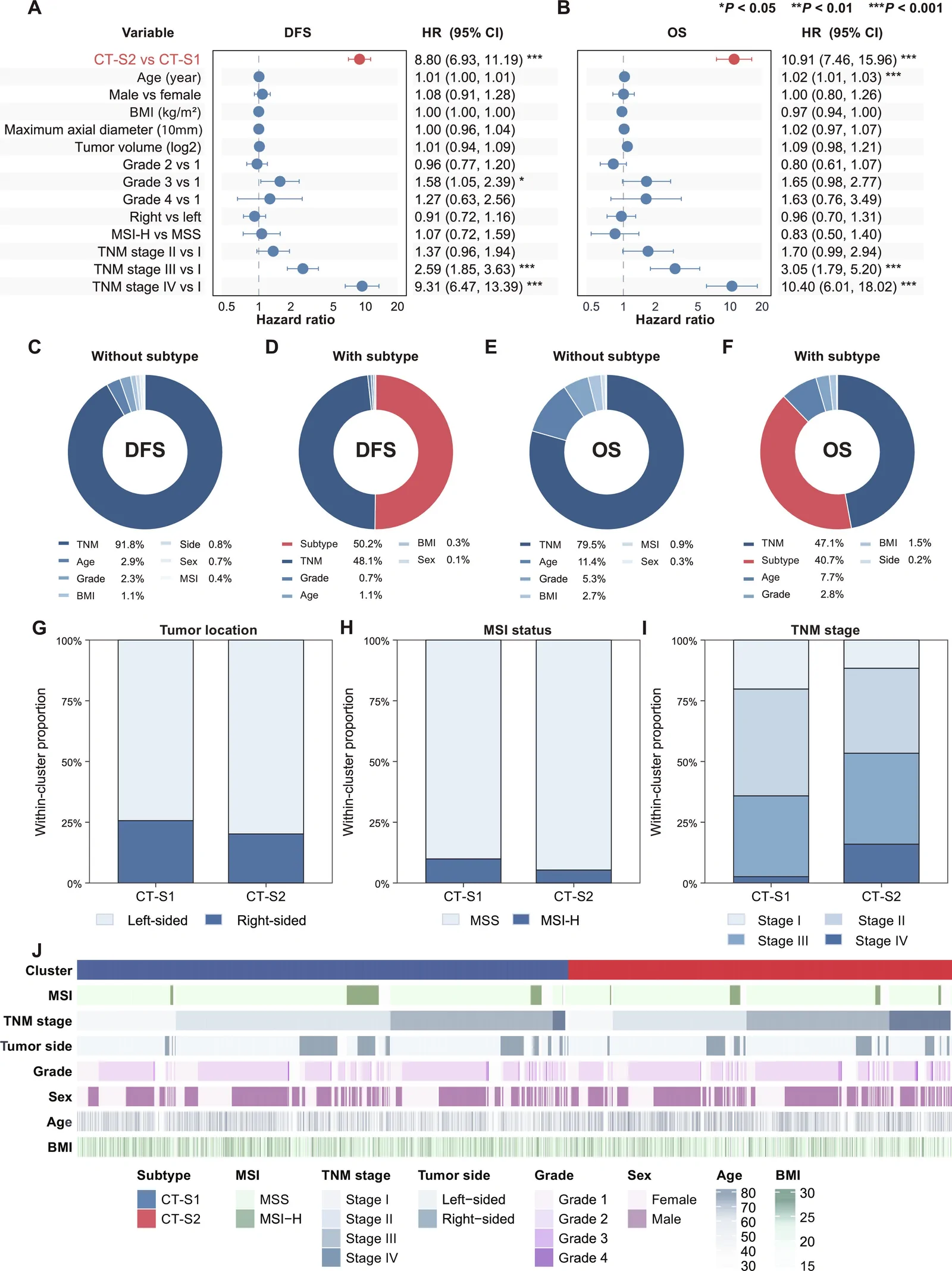
Clinical relevance and prognostic value of survival-informed computed tomography (CT) phenotypes. (A and B) Multivariable Cox forest plots for disease-free survival (DFS) (A) and overall survival (OS) (B) including CT phenotype, clinicopathologic covariates, region of interest (ROI)-derived maximum axial diameter per 10-mm increase, and tumor volume per doubling. (C to F) Relative contribution of clinical factors before and after adding CT phenotype for DFS (C and D) and OS (E and F). (G to I) Within-cluster proportion plots for tumor location (G), microsatellite instability (MSI) status (H), and TNM stage (I). (J) Clinical overview heatmap showing CT phenotype, MSI status, TNM stage, tumor side, grade, sex, age, and body mass index (BMI) across SYSU6H cohort 2.

The clinical composition of the 2 CT phenotypes was then examined (Fig. 3G to J). CT-S2 contained a higher proportion of advanced-stage disease, including stage IV tumors, and showed differences in MSI status and tumor sidedness. A clinical feature heatmap summarized relationships among CT phenotype, MSI status, TNM stage, tumor side, grade, sex, age, and BMI. These associations indicate that the CT phenotypes are embedded in recognizable clinical patterns, while adjusted survival analyses support their prognostic relevance beyond standard clinical variables. Consistent with this interpretation, stage-stratified analyses showed that CT-S2 remained associated with worse DFS and OS within TNM stages I to IV, while the proportion of CT-S2 increased with advancing stage (Fig. S5).

### Survival-informed CT phenotypes are associated with molecular programs and CMS composition

To explore the molecular correlates of the survival-informed CT phenotypes, we evaluated mutation, CMS, transcriptomic, pathway, proliferation, and predicted drug-sensitivity features (Fig. 4). A phenotype-faceted mutation waterfall plot summarized the top mutated genes in samples with available mutation data. CT-S1 showed a modestly higher overall mutation burden than CT-S2 (median mutation count, 99.0 versus 93.5; Wilcoxon *P* = 0.013). Several frequently altered genes were more common in CT-S1, including MUC16, RYR1, RYR2, ZFHX4, FAT4, FBXW7, LRP1B, CSMD1, CCDC168, and FLG (Fig. 4A). This pattern is biologically plausible because MUC16, FAT4, and RYR2 alterations have been linked in prior studies to immune activity, tumor mutation burden, or outcome-related phenotypes in CRC or other solid tumors, whereas FBXW7 and LRP1B are established cancer-related genes connected to cell cycle, Wnt/Notch, genomic instability, or immunotherapy-relevant biology [14–18]. Together with the MSI/MMR and pathway results below, the mutation profile is consistent with the interpretation that CT-S1 and CT-S2 are associated with different exploratory molecular patterns.

**Fig. 4.**
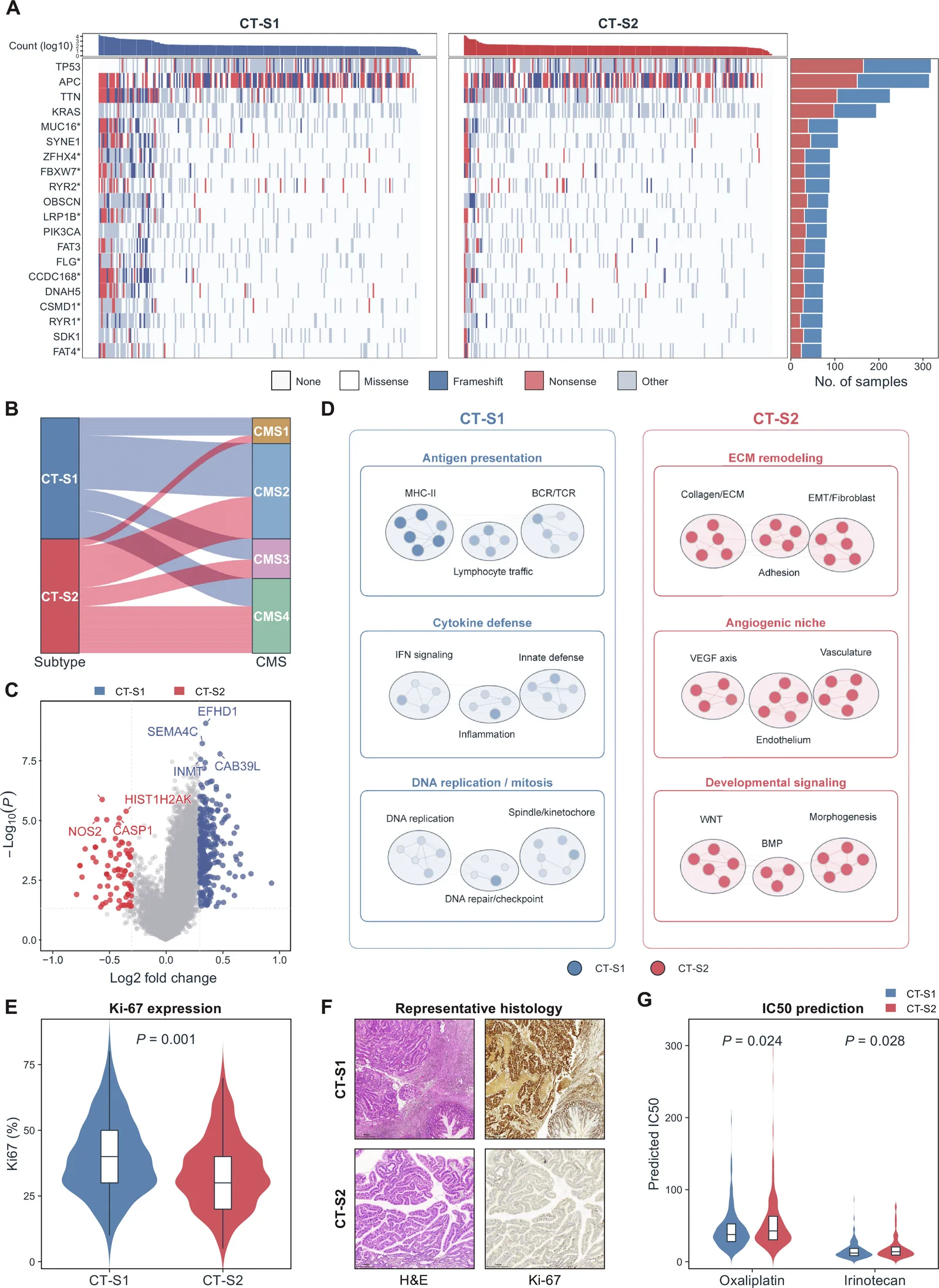
Molecular and biological correlates of survival-informed computed tomography (CT) phenotypes. (A) Faceted mutation waterfall plot showing the top 20 mutated genes by CT phenotype. Asterisks indicate nominally significant differences in mutation frequency between CT-S1 and CT-S2 by two-sided Fisher’s exact tests (*P* < 0.05, without adjustment for multiple comparisons). (B) Sankey diagram linking CT phenotype to consensus molecular subtype (CMS) classification. (C) Volcano plot of differential gene expression between CT phenotypes. (D) Biological-process pathway-network visualization summarizing phenotype-associated programs. (E) Ki-67 expression distribution by CT phenotype. (F) Representative hematoxylin and eosin (H&E) and Ki-67 immunohistochemistry images by CT phenotype. (G) Computationally predicted half-maximal inhibitory concentration (IC50) distributions for oxaliplatin and irinotecan by CT phenotype. *P* values are shown in the panels. MHC-II, major histocompatibility complex class II; BCR, B-cell receptor; TCR, T cell receptor; ECM, extracellular matrix; EMT, epithelial-mesenchymal transition; IFN, interferon; VEGF, vascular endothelial growth factor; WNT, Wingless-related integration site; BMP, bone morphogenetic protein.

CMS mapping showed distinct molecular subtype composition between CT-S1 and CT-S2 (Fig. 4B). CT-S1 contained a higher proportion of CMS2 tumors, whereas CT-S2 was enriched for CMS4-like tumors (CMS4: 40.8% in CT-S2 versus 23.2% in CT-S1). Given the known association of CMS4 with mesenchymal and stromal programs [4–6,19,20], this enrichment provides a plausible molecular context for the adverse prognosis of CT-S2. Molecular and pathology analyses were performed in the subsets with available assay data.

To assess potential selection associated with assay availability, we compared baseline characteristics between each available assay subset and the remainder of SYSU6H cohort 2 (TableS6). The groups were broadly comparable for several characteristics, although some differences were observed across assay subsets. We therefore interpret the molecular and pathology findings as exploratory associations and consider potential selection related to assay availability when discussing these results.

Differential expression and pathway-network analyses further linked the CT phenotypes to biological programs relevant to prognosis, therapy response, and the tumor microenvironment (Fig. 4C and D). The displayed network organizes phenotype-associated biological-process terms into functional modules related to prognosis, chemotherapy response, and immune microenvironment [19,21–23]. Complementary pathway and marker analyses showed higher WNT/β-catenin, Notch, TGF-β, myogenesis, and Hedgehog pathway scores in CT-S2, whereas CT-S1 showed higher interferon-response and allograft-rejection pathway scores and immune-marker expression (Fig. S6). Together, these findings suggest that CT-derived phenotypes may reflect broader transcriptomic and microenvironment-associated patterns beyond isolated imaging variation.

We also assessed clinically interpretable biological readouts. Ki-67 expression was higher in CT-S1 than in CT-S2 (median 40% versus 30%, *P* = 0.001; Fig. 4E), with representative hematoxylin and eosin and Ki-67 immunohistochemical staining images shown in Fig. 4F [24]. Computational drug-sensitivity analysis showed lower predicted half-maximal inhibitory concentration (IC50) values in CT-S1 for oxaliplatin and irinotecan (oxaliplatin median 37.66 versus 42.72, *P* = 0.024; irinotecan median 11.95 versus 13.83, *P* = 0.028; Fig. 4G) [25–28]. These findings are consistent with the possibility that CT-S1 may reflect a more proliferative imaging phenotype with greater chemotherapy-related transcriptional susceptibility, although the predicted IC50 values reflect computationally inferred drug sensitivity rather than observed clinical response.

### A phenotype-assignment model enables internal and external cohort evaluation

Consensus clustering was first used to derive CT-S1 and CT-S2 in SYSU6H cohort 2. The resulting cluster labels were then used as the only supervised targets to train a separate phenotype-assignment model for direct assignment in new patients. This model accepted CRCFound-only features, PyRadiomics-only features, or fused features (Fig. 5A). During training, modality dropout exposed the model to single-branch and fused inputs, and a feature-gating module with a multilayer perceptron (MLP) classifier produced phenotype probabilities.

**Fig. 5.**
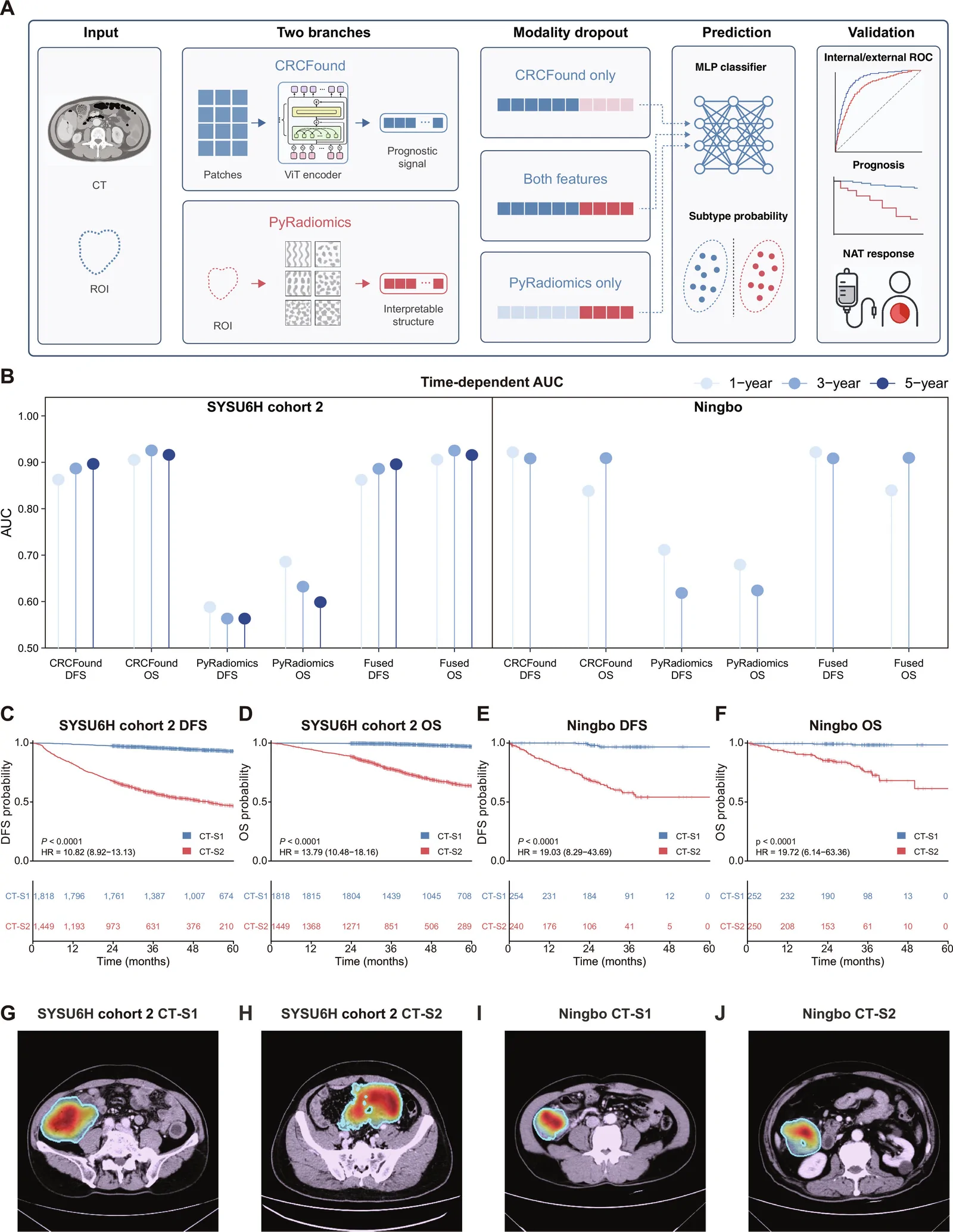
Phenotype-assignment modeling and evaluation across cohorts. (A) Workflow of the dual-branch phenotype-assignment model. CT-S1 and CT-S2 labels derived by consensus clustering in SYSU6H cohort 2 served as the supervised training targets; no survival outcome was used during assignment-model training. CRCFound and PyRadiomics encoders produce modality-specific feature vectors. Modality dropout trains the model to accept CRCFound-only, PyRadiomics-only, or fused inputs. A shared predictor outputs phenotype probabilities. (B) Time-dependent areas under the curve (AUCs) for 1-, 3-, and 5-year disease-free survival (DFS) and overall survival (OS) discrimination using CRCFound-only, PyRadiomics-only, and fused phenotype-assignment outputs in SYSU6H cohort 2 and Ningbo. Endpoint-specific unavailable AUCs are omitted. (C to F), Kaplan–Meier curves for predicted phenotype groups in SYSU6H cohort 2 DFS (C), SYSU6H cohort 2 OS (D), Ningbo DFS (E), and Ningbo OS (F). (G to J) Representative region of interest (ROI)-focused computed tomography (CT) attribution heatmaps for SYSU6H cohort 2 CT-S1 (G), SYSU6H cohort 2 CT-S2 (H), Ningbo CT-S1 (I), and Ningbo CT-S2 (J).

Downstream time-dependent area under the curve (AUC) analysis showed that CRCFound and fused inputs provided strong survival discrimination among assigned phenotype groups in SYSU6H cohort 2 and Ningbo analyses (Fig. 5B). In the SYSU6H cohort 2 (*N* = 3,267), CRCFound achieved 1-, 3-, and 5-year AUCs of 0.869, 0.887, and 0.892 for DFS and 0.902, 0.919, and 0.899 for OS. Fused inputs showed similar values, with 1-, 3-, and 5-year AUCs of 0.869, 0.886, and 0.891 for DFS and 0.903, 0.919, and 0.899 for OS. PyRadiomics-only performance was lower but remained above chance for most endpoints. In Ningbo cohort 1 (*N* = 509), CRCFound and fused phenotype-assignment outputs maintained high 1- and 3-year AUCs for DFS and OS; 5-year estimates were omitted where follow-up support was insufficient. Endpoint-specific AUC calculations used patients with complete endpoint and time data (Ningbo DFS, *N* = 494; Ningbo OS, *N* = 502).

Assigned phenotype groups showed consistent survival separation in both internal and external cohorts (Fig. 5C to F). In SYSU6H, the assigned high-risk phenotype was associated with worse DFS (HR = 10.82, 95% CI 8.92 to 13.13) and OS (HR = 13.79, 95% CI 10.48 to 18.16). In Ningbo cohort 1, assigned phenotypes also separated DFS (HR = 19.03, 95% CI 8.29 to 43.69) and OS (HR = 19.72, 95% CI 6.14 to 63.36). Representative CT attribution heatmaps localized model attention around tumor regions in selected cases, providing image-level plausibility for the learned representation (Fig. 5G to J). Because the study is retrospective, these findings are presented as a cohort-level phenotype-assignment evaluation and require independent prospective testing before clinical implementation.

Calibration of 3-year event probabilities was generally close to the ideal line in SYSU6H cohort 2, whereas deviations were observed in some higher-risk strata of the Ningbo evaluation (Figs. S7 and S8).

For the incremental 3-year evaluation, we compared a clinical base Cox model including age, sex, grade, TNM stage, tumor side, and MSI status with the same model plus the locked CT phenotype. Complete-case analyses included 2,657 patients for both endpoints in SYSU6H cohort 2 and 400 patients for DFS and 404 patients for OS in Ningbo cohort 1. In a conditional 5-fold cross-validated evaluation of the Cox layer using fixed phenotype labels in SYSU6H cohort 2, adding CT phenotype increased the inverse probability of censoring-weighted (IPCW) AUC from 0.731 to 0.855 for DFS and from 0.793 to 0.894 for OS, while reducing the Brier score from 0.130 to 0.108 and from 0.067 to 0.059, respectively. In Ningbo external evaluation, the corresponding AUCs increased from 0.805 to 0.902 for DFS and from 0.758 to 0.858 for OS, and the Brier scores decreased from 0.143 to 0.105 and from 0.099 to 0.085. Continuous net reclassification improvement and integrated discrimination improvement point estimates were positive in all 4 analyses, and the augmented model showed higher net benefit across most evaluated threshold probabilities (Figs. S7 and S8 and Table S6). At 3 years, the estimated probabilities of remaining uncensored in Ningbo were 0.318 for DFS and 0.348 for OS, indicating limited follow-up support for the external estimates.

### Survival-informed CT phenotypes show potential relevance in a neoadjuvant-treatment cohort

Finally, we evaluated phenotype relevance in the SYSU6HNAT neoadjuvant-treatment cohort (Fig. 6). Assigned CT phenotypes stratified survival in this cohort. Time-dependent receiver operating characteristic (ROC) analysis showed high discrimination for DFS (1-, 3-, and 5-year AUCs of 0.846, 0.881, and 0.923) and OS (AUCs of 0.826, 0.850, and 0.891; Fig. 6A and C). Kaplan–Meier analysis showed separation between phenotype groups for both DFS and OS (DFS HR = 23.37, 95% CI 11.50 to 47.49; OS HR = 32.16, 95% CI 10.16 to 101.80; both *P* < 0.0001; Fig. 6B and D).

**Fig. 6.**
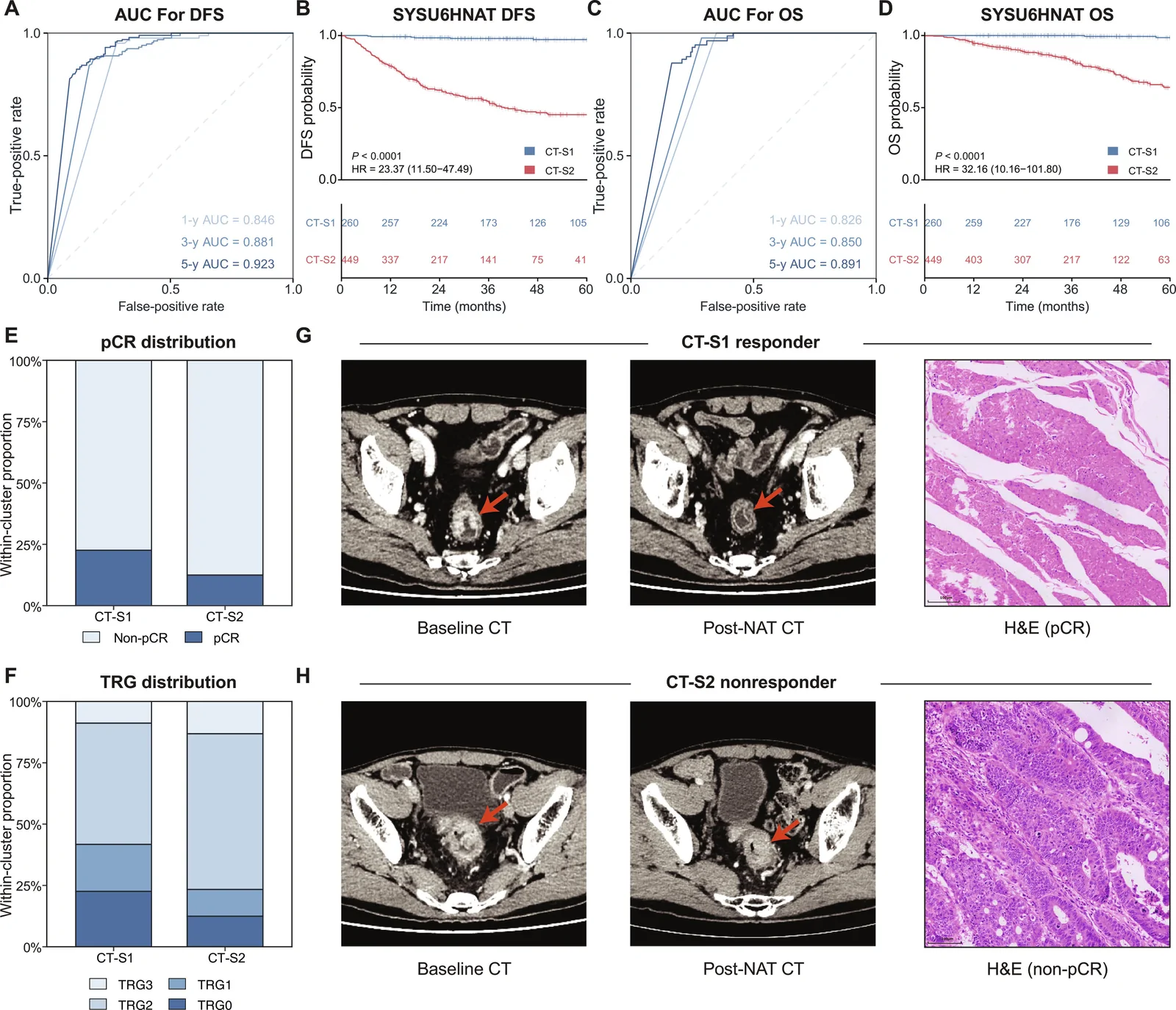
Prognostic and treatment-response relevance in SYSU6HNAT. (A and C) Time-dependent receiver operating characteristic (ROC) curves for disease-free survival (DFS) (A) and overall survival (OS) (C) in the SYSU6HNAT neoadjuvant-treatment cohort. (B and D) Kaplan–Meier curves for DFS (B) and OS (D) by computed tomography (CT) phenotype in SYSU6HNAT. (E) Within-cluster pathological complete response (pCR) distribution. (F) Within-cluster tumor regression grade (TRG) distribution. (G and H) Representative pre- and postneoadjuvant CT and hematoxylin-and-eosin (H&E) pathology examples by phenotype/response category. In the SYSU6HNAT cohort, CT-S1 showed higher pCR and TRG0 proportions than CT-S2 and a lower TRG3 proportion, compatible with an association between CT-S1 and more favorable pathological response in this retrospective cohort.

CT phenotype was also associated with pathological response. In the SYSU6HNAT cohort, CT-S1 had a higher pCR rate than CT-S2 (59/260, 22.7% versus 56/449, 12.5%; Fisher’s exact test, *P* < 0.001; Fig. 6E). TRG distribution was more favorable in CT-S1, with a higher TRG0 proportion and lower TRG3 proportion than CT-S2 (TRG0: 22.7% versus 12.5%; TRG3: 8.8% versus 13.1%; chi-square test, *P* < 0.001; Fig. 6F). Representative pre- and postneoadjuvant CT and pathology examples across phenotype/response categories are shown in Fig. 6G and H. Additional baseline comparisons in SYSU6HNAT showed a significant difference in carcinoembryonic antigen distribution between CT phenotypes, whereas grade, CA19-9, and MMR status showed descriptive phenotype-related trends without reaching statistical significance after excluding unknown categories from the displayed proportions (Fig. S9). These findings indicate a retrospective association between CT phenotype and pathological response, supporting further validation of CT phenotype as a pretreatment imaging biomarker in neoadjuvant-treated CRC.

## Discussion

We developed an integrated CT phenotyping framework for CRC that combines foundation-model features with handcrafted radiomic descriptors from the same tumor region. The fused representation identified 2 survival-informed CT imaging phenotypes with distinct survival, clinical composition, molecular correlates, and neoadjuvant-treatment associations. These findings suggest that routine venous-phase CT imaging can support a noninvasive imaging-based phenotyping framework associated with clinically and molecularly relevant programs.

This work addresses a central translational gap in CRC heterogeneity research. Molecular subtyping has clarified important biological states [4–6], but sequencing-based classification is not always available before treatment and is difficult to implement at the population scale. Radiomics provides interpretable quantitative descriptors of tumor morphology and texture [7–11], whereas foundation-model features can capture higher-capacity image representations learned from large-scale CT data [13]. By integrating both sources, this framework aims to retain the interpretability of radiomics while using deep features to improve imaging phenotype derivation. The result is not limited to a scalar prognostic estimate but instead provides a survival-informed imaging phenotyping framework that can be examined across clinical and molecular dimensions.

The clustering step was retained to produce discrete patient groups for clinicopathologic and molecular characterization, complementing rather than replacing continuous risk prediction. Within this prognosis-oriented framework, the resulting groups are interpreted as survival-informed imaging phenotypes with clinically interpretable and exploratory biological correlates.

The survival analyses support the clinical relevance of the CT phenotypes. In SYSU6H cohort 2, CT-S2 was associated with worse DFS and OS after adjustment for clinicopathologic variables, ROI-derived tumor volume, and maximum axial diameter. In Ningbo cohort 1, the association remained in a reduced multivariable model including age, sex, grouped TNM stage, maximum axial diameter, and tumor volume. CT-S2 also contained more advanced-stage and larger tumors; accordingly, CT phenotype should be considered together with anatomical stage and tumor size. Its persistent prognostic association after multivariable adjustment and within TNM strata suggests that CT phenotype may add a noninvasive risk dimension to standard staging and clinicopathologic assessment. Because tumor volume and maximum axial diameter were derived from the same ROI as the imaging features, tumor size may represent both a potential confounder and a component of the imaging phenotype. We therefore interpret the size-adjusted models as sensitivity analyses testing robustness to measured tumor size, rather than as a definitive separation of tumor size from the phenotype. Prospective validation is required to establish clinical utility.

CRCFound and PyRadiomics contributed complementary forms of information. Handcrafted radiomics quantifies a predefined set of shape, intensity, and texture statistics, whereas CRCFound was pretrained on large-scale 3-dimensional (3D) CT data and can encode multiscale spatial context, intratumoral heterogeneity, and relationships between the tumor and surrounding tissues within distributed deep representations [7–13]. Subsequent survival adaptation may further align these representations with outcome-associated imaging patterns. Deep learning approaches in radiology and cancer imaging can capture complex image information across distributed representations that is not fully summarized by predefined handcrafted descriptors [29]. The stronger survival discrimination observed for CRCFound may reflect both its broader representation space and survival-informed adaptation. Fusion combined this representation capacity with the interpretability of radiomic features, consistent with the broader rationale for combining complementary imaging modalities in precision imaging [30].

The biological analyses provide exploratory molecular context for the imaging phenotypes. CT-S2 was enriched for CMS4-like tumors and stromal/extracellular-matrix programs, consistent with a mesenchymal and microenvironment-rich state associated with adverse outcome [4–6,19,20]. In contrast, CT-S1 showed higher Ki-67 expression [24], a modestly higher mutation burden, more frequent alterations in genes linked to immune or genomic-instability phenotypes [14–18], and lower computationally predicted IC50 values for oxaliplatin and irinotecan [25–28]. Taken together, these observations provide associative links between CT phenotype and tumor proliferation, stromal remodeling, immune contexture, and treatment-relevant transcriptional programs, while remaining distinct from direct mechanistic evidence [19–23,31].

The phenotype-assignment results provide a practical bridge between survival-informed imaging phenotype derivation and evaluation in additional cohorts. After phenotype derivation by clustering in SYSU6H cohort 2, a separate dual-branch model was trained using the cluster-derived CT-S1 and CT-S2 labels and then applied to CRCFound-only, PyRadiomics-only, or fused inputs; the assigned phenotypes reproduced prognostic separation in internal and Ningbo external evaluation cohorts. This assignment step would be needed for future testing because it avoids rerunning clustering for every new case, and the modality-flexible design may be useful when one feature source is unavailable. These results should nevertheless be interpreted as a retrospective internal and external cohort evaluation rather than definitive clinical validation [32].

The SYSU6HNAT analysis explored whether the CT phenotype was associated with outcomes in a neoadjuvant-treatment setting. CT-S1 showed more favorable pCR and TRG distributions than CT-S2, indicating a retrospective association between pretreatment imaging phenotype and pathological response [33–35]. This pattern is concordant with the higher Ki-67 and lower computationally predicted chemotherapy IC50 profile observed in CT-S1 [24–28]. However, phenotype-guided treatment selection was not tested prospectively, and the current data should be used to motivate validation studies rather than to support immediate changes in treatment strategies.

Importantly, this study goes beyond simply proposing a high-performance prognostic model; it establishes a noninvasive stratification framework that integrates conventional CT, baseline model characterization, interpretable radiomic features, and the multiomic biological status of CRC. In a large-scale, multicenter cohort, this framework identified a reproducible high-risk CT phenotype that remained associated with outcomes after adjustment for TNM stage and MSI status and was linked to CMS4-like composition, stromal remodeling, and retrospective pathological-response patterns, providing a scalable CT-based stratification pathway for CRC patients who cannot routinely undergo molecular sequencing. Furthermore, we developed a flexible classification predictor that can operate with or without PyRadiomics features, supporting evaluation of deployment feasibility in future prospective studies. The main contribution of this study is a noninvasive CT-based phenotyping framework that links foundation-model feature capacity, radiomic interpretability, prognosis, and exploratory biological context. The findings may help prioritize future validation of imaging-defined CRC phenotypes, although prospective studies are required before phenotype-guided clinical decisions can be considered.

This study has several limitations. First, the development and validation of the CT phenotype framework proposed in this study were both based on retrospective samples. Prospective studies are needed in the future to further confirm the clinical utility and generalizability of this phenotype system. Second, all feature-selection thresholds and preprocessing parameters used for phenotype derivation were determined using the full SYSU6H cohort 2 before internal prognostic evaluation; therefore, internal performance estimates may be optimistic. A separately trained phenotype-assignment model reproduced prognostic separation in the independent external cohort without retraining or tuning, but prospective validation remains necessary. Third, the imaging phenotypes were developed for prognostic stratification; therefore, the observed molecular and therapeutic associations should be regarded as exploratory and require independent validation. Molecular profiling was incomplete, and the available assay subsets differed from the remainder of the cohort in several clinical characteristics, so selection related to assay availability cannot be excluded. Fourth, heterogeneity in imaging acquisition, ROI annotation, molecular profiling, and follow-up across participating cohorts may have influenced the reported effect sizes despite standardized preprocessing and external validation. Future prospective studies with standardized imaging protocols, comprehensive molecular characterization, and longer follow-up will be important to confirm the robustness and clinical utility of this framework. Overall, integrated CT foundation-model and radiomic phenotyping identified clinically relevant, survival-informed CRC imaging phenotypes and generated biologically plausible hypotheses for future investigation.

## Materials and Methods

### Study design, ethics, and cohorts

This retrospective multicohort study was designed to derive survival-informed CT imaging phenotypes, interpret their exploratory biological correlates, and evaluate their relevance in internal, external, and neoadjuvant-treatment settings. The study was approved by the institutional ethics committee (approval No. 2026ZSLYEC-300, KY2026SL237-01), and informed consent was waived because of the retrospective design and use of deidentified data. Reporting followed observational and prediction-model reporting principles where applicable [36]. The 4 cohorts were established independently according to project role and data availability, with no patient overlap; specifically, SYSU6H cohorts 1 and 2 were completely nonoverlapping patient cohorts. SYSU6H cohort 1 was a CT-only cohort without matched tissue samples and was used for CRCFound fine-tuning (*N* = 902; 2011 May 1 to 2020 May 30). SYSU6H cohort 2 was an independent multimodal cohort with linked tissue and omics resources and was used for imaging phenotype derivation and survival analyses (*N* = 3,267; 2008 January 1 to 2020 April 30). Because of sequencing cost and incomplete assay availability, molecular analyses were restricted to the available assay subsets. Ningbo cohort 1 was an institutionally independent external evaluation cohort that was not used for model training or parameter tuning (*N* = 509; 2020 January 1 to 2022 October 31). SYSU6HNAT was a separate cohort of patients receiving neoadjuvant treatment and was used for treatment-response evaluation (*N* = 709; 2012 November 1 to 2024 April 30). Detailed cohort characteristics are provided in Tables S1 and S2.

CRCFound representation adaptation was restricted to SYSU6H cohort 1. No patients or scans from SYSU6H cohort 2, Ningbo cohort 1, or SYSU6HNAT, and no preprocessing estimates or model weights derived from those cohorts, entered the adaptation process. The adapted representation was subsequently used for feature extraction in SYSU6H cohort 2 and as an input representation for phenotype assignment in the independent evaluation cohorts.

Eligible patients had histopathologically confirmed CRC and available baseline venous-phase contrast-enhanced CT images before the relevant treatment or outcome assessment. General exclusion criteria included unavailable or technically unusable CT images, unidentifiable primary tumor, unsuccessful ROI matching or feature extraction, duplicate or unresolved identifiers, and no valid outcome or follow-up information for the analysis of interest. Patients with valid follow-up who were subsequently lost to follow-up were retained and censored at the date of their last known follow-up. DFS was analyzed as the interval from surgery to the first documented recurrence, metastasis, or death. OS was analyzed as the interval to death from any cause or last follow-up. For SYSU6HNAT, pCR and TRG were used as pathological response endpoints after neoadjuvant treatment [33–35].

### CT acquisition, preprocessing, and ROI annotation

Center-specific scanner and acquisition distributions are reported in Table S2. All analyzed CT images were venous-phase contrast-enhanced scans. Median slice thickness was 3.8 mm (IQR, 3.0 to 3.8 mm) at SYSU6H and 2.0 mm (IQR, 2.0 to 2.0 mm) at Ningbo. No scanner-specific statistical harmonization such as ComBat was applied. Instead, all images underwent the same prespecified spatial and intensity preprocessing, and transportability was evaluated in the institutionally independent Ningbo cohort.

CT series were ordered by slice position or instance number and converted to volumetric images while preserving spatial metadata. CT volumes were resampled to 1.0 × 1.0 × 3.0 mm using linear interpolation. For cohorts with manual tumor ROIs, masks were resampled using nearest-neighbor interpolation, and CT volumes were cropped or padded around the ROI center to 32 × 256 × 256 voxels. For the PyRadiomics branch, these ROI-centered CT volumes were windowed at a level of 35 HU and width of 300 HU and used directly for radiomic feature extraction. For the CRCFound branch in these cohorts, a branch-specific copy of the same windowed volume was linearly scaled to [0,1]. Because manual tumor ROIs were unavailable for SYSU6HNAT, this cohort was evaluated through the CRCFound-only pathway: Resampled CT volumes were geometrically center-cropped or padded to the same target size, windowed identically, and scaled to [0,1]; PyRadiomics features and ROI-derived tumor-size measurements were not calculated.

Tumor ROIs in the ROI-based cohorts were manually delineated by junior radiologists or physicians (with 3 to 5 years of experience in gastrointestinal oncology) and revised by senior radiologists (with 18 to 25 years of subspecialty experience in gastrointestinal oncology) using the open-source software ITK-SNAP (version 3.8.0, www.itksnap.org). ROIs were annotated along visible tumor margins on each transverse section until the entire tumor was completely covered. Necrotic regions within the tumor were included, whereas intraluminal gas or fecal material, uninvolved bowel lumen, and peritumoral fat were excluded. The CT preprocessing workflow and PyRadiomics configuration are publicly available in the CTcluster repository (https://github.com/LidocaineQ/CTcluster/tree/main/pyradiomics_extraction).

For interobserver reproducibility, 30 cases were randomly selected from SYSU6H cohort 2 and independently redelineated by an additional radiologist (with 13 years of experience in gastrointestinal oncology). Spatial agreement was assessed using DSC, Jaccard index, 95th-percentile Hausdorff distance, and average symmetric surface distance. Agreement for tumor volume, maximum axial diameter, and PyRadiomics features was assessed using ICC (2,1), a 2-way random-effects, absolute-agreement, single-measurement model (Fig. S1 and Table S3).

### CRCFound feature extraction and survival fine-tuning

CRCFound was used as the deep CT foundation-model branch [13]. The model used a 3D vision-transformer architecture with a 256-pixel image size, 32 frames, 16-pixel image patch size, 16-frame patch size, and a 1,024-dimensional latent representation. Survival fine-tuning was performed for DFS and OS using Cox loss exclusively in SYSU6H cohort 1, and the adaptation settings were determined without using any data from SYSU6H cohort 2 or the external cohorts. Training used the AdamW optimizer, cosine annealing learning-rate scheduling, and alternating DFS and OS loaders. Most backbone parameters were frozen, whereas the MLP head, adapter blocks, and prompt parameters were trainable. Deep embeddings were extracted from the best survival fine-tuned model using the global representation, yielding 1,024-dimensional CRCFound features.

### PyRadiomics feature extraction

Handcrafted radiomic features were extracted using PyRadiomics v3.0.1 [10]. Features were extracted from the window-clipped CT volumes with a fixed bin width of 25 HU. The [0,1]-scaled CRCFound input was not used for PyRadiomics extraction. Extraction used label 1 and the configuration publicly available in the CTcluster repository (https://github.com/LidocaineQ/CTcluster/tree/main/pyradiomics_extraction). Original, wavelet, logarithm, exponential, square, square root, gradient, local-binary-pattern, and 2D/3D image types were enabled together with shape, first-order, GLCM, GLRLM, GLSZM, GLDM, and NGTDM feature classes, following radiomics standardization principles [11].

### Survival-informed feature selection, fusion, and imaging phenotype derivation

Survival-informed imaging phenotype derivation was performed in SYSU6H cohort 2 (*N* = 3,267). Numeric features were filtered to remove constant features and features with at least 20% missing values. Survival outcomes from SYSU6H cohort 1 were used during CRCFound representation adaptation, whereas survival outcomes from SYSU6H cohort 2 were used for modality-specific univariable Cox feature screening. The subsequent clustering algorithm received only the selected imaging features and did not receive event labels or survival times. This step reduced feature dimensionality before downstream dimensionality reduction and clustering, limiting the inclusion of noisy features.

Selected features were processed by median imputation, standard scaling, and quantile transformation to a normal distribution. Each modality was reduced by principal component analysis. Modality-specific similarity networks were constructed from Euclidean distances using a Gaussian kernel and K-nearest-neighbor sparsification. The networks were integrated using a similarity-network-fusion-like weighted iterative procedure. Consensus clustering combined spectral clustering, k-means, and Gaussian-mixture assignments across repeated runs, accumulated in a consensus matrix, and applied agglomerative clustering to 1 minus the consensus matrix. Candidate cluster numbers from *k* = 2 to *k* = 8 were evaluated using silhouette coefficients, minimum cluster proportion, clustering stability, and survival separation. The final *k* = 2 configuration used a higher weight for CRCFound than for PyRadiomics and generated CT-S1 (*n* = 1,835) and CT-S2 (*n* = 1,432). All feature-selection thresholds, preprocessing estimates, dimensionality-reduction settings, modality-integration settings, and clustering hyperparameters used for phenotype derivation were determined using the complete SYSU6H cohort 2 before internal prognostic evaluation. Therefore, prognostic performance based on the cluster-derived phenotypes in this cohort may be optimistic and may not fully represent prospective performance. These cohort 2-derived settings did not enter CRCFound representation adaptation. Phenotype assignment in additional cohorts was handled in a subsequent, separate modeling stage.

For sensitivity analysis, feature processing and consensus clustering were repeated without the modality-specific univariable Cox feature-screening step. The resulting *k* = 2 solution was evaluated using Harrell C-index for DFS and OS and the number of Hallmark pathways differing between groups at BH-FDR < 0.05; binary cluster labels were oriented to the prognostic direction for C-index reporting, and pathway counts were compared with those of the principal CT phenotypes (Fig. S3 and Table S4).

### Survival, multivariable Cox, and clinical contribution analyses

DFS and OS were analyzed using Kaplan–Meier estimates and log-rank tests. HRs were estimated using Cox proportional-hazards models. Survival analyses retained patients with complete event and time information and positive follow-up time. In SYSU6H cohort 2, principal multivariable Cox models included CT phenotype, TNM stage, MSI status, tumor side, grade, BMI, sex, age, maximum axial diameter per 10-mm increase, and log2-transformed tumor volume interpreted per doubling. Tumor volume was calculated from positive-mask voxel count and voxel volume; maximum axial diameter was the largest 2-dimensional Feret diameter across axial ROI slices. For external evaluation in Ningbo cohort 1, an unpenalized complete-case Cox model included CT phenotype, age, sex, TNM stage grouped as I-II versus III-IV, maximum axial diameter per 10-mm increase, and log2-transformed tumor volume interpreted per doubling. For the tumor-size sensitivity analysis, each endpoint was refitted on an identical complete-case set with and without the 2 tumor-size covariates. The multivariable models without tumor-size covariates were summarized in Fig. S4C and D, and the paired CT-S2 HRs with and without tumor-size adjustment were summarized in Table S5.

Clinical contribution analyses compared multivariable clinical models before and after adding the CT phenotype. For each model, the relative contribution of included factors was summarized from model-based prognostic information and normalized to sum to 100% within the displayed panel. These analyses were used to illustrate the contribution of CT phenotype alongside TNM stage and other covariates, not to infer causality or replace anatomical staging.

### Molecular, transcriptomic, pathway, Ki-67, and drug-sensitivity analyses

Mutation analysis summarized phenotype-specific mutation frequencies in samples with available mutation data using standard somatic-mutation visualization procedures [37]. Differential expression analysis used limma with linear-model fitting, contrast fitting, empirical Bayes moderation, and FDR-adjusted output [38]. Hallmark pathway analysis used limma on the Hallmark pathway matrix [39]. Biological-process enrichment visualization organized phenotype-associated terms into literature-relevant programs related to prognosis, chemotherapy response, and immune microenvironment, with gene-set interpretation supported by clusterProfiler [40]. Effective sample sizes were 481 for transcriptome/CMS analyses, 462 for mutation analyses, and 272 for Ki-67.

To assess potential selection associated with assay availability, patients with available transcriptome/CMS, mutation, or Ki-67 data were separately compared with the remainder of SYSU6H cohort 2 across clinical and imaging baseline variables. Continuous variables were compared using 2-sided Mann–Whitney U tests, and categorical variables using chi-square or Fisher’s exact tests, as appropriate. Benjamini–Hochberg-adjusted *P* values and absolute standardized mean differences were reported within each assay panel, with available-case denominators retained for each variable (Table S6).

Ki-67 analysis used available pathology records and compared the 2 CT phenotypes using the Wilcoxon rank-sum test [24]. Representative Ki-67 immunohistochemistry images were displayed by CT phenotype. Computational drug-sensitivity analysis estimated IC50 values from tumor transcriptomic profiles using an oncoPredict/GDSC2-based workflow with empirical-Bayes batch correction, phenotype power transformation, removal of low-variance genes, and minimum sample requirements [25–28]. IC50 values were analyzed on the log2 scale, with Wilcoxon tests for *k* = 2 comparisons. These results are described as computationally predicted IC50 rather than observed clinical treatment response.

### Flexible dual-branch phenotype-assignment model

The CT-S1 and CT-S2 labels obtained by consensus clustering in SYSU6H cohort 2 were used as the only supervised targets to train a flexible phenotype-assignment model from CRCFound-only, PyRadiomics-only, or fused inputs. Survival outcomes, event indicators, and survival times were not used for feature screening, model selection, or training during this modeling stage. Radiomic and deep features were processed with imputation, supervised feature screening against the cluster-derived labels, and standard scaling before entering modality-specific branches. The predictor used branch-specific encoders, a shared latent representation, modality dropout during training, and an MLP classifier to output phenotype probabilities. Modality dropout exposed the model to joint-input, CRCFound-only, and PyRadiomics-only settings so that phenotype assignment could still be performed when one feature source was unavailable. Model selection used stratified train-validation splits based on the cluster-derived labels. The trained phenotype-assignment model was fixed before application to Ningbo cohort 1 and SYSU6HNAT, with no retraining or tuning in either evaluation cohort. Final AUC values were calculated using the 3,267-patient SYSU6H cohort 2 and endpoint-complete records for each endpoint.

### SYSU6HNAT neoadjuvant-treatment analysis

The SYSU6HNAT cohort (*N* = 709 patients) was used to evaluate CT phenotype association with DFS, OS, pCR, and TRG after standard neoadjuvant treatment [33,34]. Neoadjuvant regimens included fluoropyrimidine-, oxaliplatin-, and irinotecan-containing chemotherapy, with radiotherapy administered in a subset of patients according to clinical practice. For patients receiving long-course chemoradiotherapy, the treatment-to-surgery interval was consistent with conventional guideline-based timing [33,34]. pCR was defined as no viable tumor in the resected primary tumor and regional lymph nodes (ypT0N0). TRG was reported using institutional coding, with TRG0 indicating complete regression and TRG3 indicating poor or no regression [35]. pCR rates were compared using Fisher’s exact test; TRG distributions were compared using chi-square tests or nonparametric tests as appropriate. Figure 6G and H contains representative pre- and postneoadjuvant CT and hematoxylin-and-eosin pathology examples.

### Statistical analysis

Continuous variables were compared using parametric or nonparametric tests as appropriate, and categorical variables using chi-square tests or Fisher’s exact tests. Time-dependent ROC analyses were used for 1-, 3-, and 5-year discrimination where follow-up support was sufficient [32]. Additional 3-year evaluation compared a clinical base Cox model including age, sex, grade, TNM stage, tumor side, and MSI status with the same model plus the locked CT phenotype. Analyses used complete cases for the included clinical variables and endpoint. Internal predictions were generated by a conditional 5-fold, event-stratified cross-validated evaluation of the Cox layer in SYSU6H cohort 2; age standardization, model fitting, and baseline-risk estimation were performed using the training folds only. The locked phenotype labels were retained, and unsupervised clustering was not repeated within each fold. External predictions were generated by models fitted in SYSU6H cohort 2 and evaluated without refitting in Ningbo cohort 1. For the 3-year incremental performance analysis, censoring was handled using IPCW, with the censoring distribution estimated using the Kaplan–Meier method and without weight truncation. Time-dependent discrimination was quantified using the IPCW area under the ROC curve, whereas overall prediction error was evaluated using the IPCW Brier score, with lower values indicating better predictive accuracy. Calibration was assessed by grouping predicted 3-year event probabilities into deciles and comparing stratum-level predicted risks with Kaplan–Meier event estimates. Clinical utility was evaluated using IPCW decision-curve analysis over threshold probabilities from 0.05 to 0.50. Net benefit was calculated as TP/*n* − FP/*n* × pt/(1 − pt), where TP and FP denote the IPCW-weighted numbers of true-positive and false-positive classifications, respectively, *n* denotes the sample size, and pt denotes the threshold probability; the clinical base model and the model additionally including CT phenotype were compared with the treat-all and treat-none strategies. Incremental reclassification was summarized using continuous net reclassification improvement, which quantified the net proportion of patients whose predicted risks moved in the correct direction among those with and without a 3-year event, and integrated discrimination improvement, which quantified the change in separation of mean predicted risks between the 2 outcome groups. For pathway-level multiple comparisons, *P* values were adjusted using the Benjamini–Hochberg procedure, and pathways with BH-FDR < 0.05 were considered significant. Harrell C-index was used to quantify survival discrimination in the feature-screening sensitivity analysis. All statistical tests were 2-sided.

## Data Availability

The data that support the findings of this study are available on request from the corresponding authors. The imaging and clinical data are not publicly available because of patient-privacy, ethical, and institutional restrictions. Access to deidentified data may be considered for scientifically appropriate purposes after approval by the relevant ethics committee and completion of a data-use agreement. Deidentified illustrative code templates for the principal computational components, together with the study-specific CT preprocessing workflow and PyRadiomics configuration, are publicly available at GitHub (https://github.com/LidocaineQ/CTcluster). Private cohort data, model weights, and cohort-specific analysis scripts are not publicly available.
