# Supplementary material for: Integrated CT Foundation Model and Radiomic Features Define Clinically and Biologically Distinct Colorectal Cancer Subtypes
Source: Research (Wash D C). 2026 Oct 6;9:1435. doi: 10.34133/research.1435 (PMC13639147; doi:10.34133/research.1435)
Supplement: Supplementary file 1 [file research.1435.f1.zip › Figs. S1 to S9.docx]

**Supplementary Figures**

This file contains 9 supplementary figures.

Supplementary Figure 1. Interobserver ROI and radiomic reproducibility

Supplementary Figure 2. Single-modality and fused phenotype analyses

Supplementary Figure 3. Sensitivity analysis with and without Cox-based feature screening

Supplementary Figure 4. Tumor size comparisons and Cox sensitivity analyses

Supplementary Figure 5. Stage-stratified clinical and survival analyses of CT phenotypes

Supplementary Figure 6. Additional molecular and pathway correlates of CT phenotypes

Supplementary Figure 7. External 3-year model evaluation in Ningbo cohort 1

Supplementary Figure 8. Internal cross-validated 3-year model evaluation in SYSU6H cohort 2

Supplementary Figure 9. Baseline clinical feature distributions in SYSU6HNAT

**Supplementary Figure 1. Interobserver ROI and radiomic reproducibility** (A), Distribution of DSC values for 30 randomly selected cases independently redelineated by an additional radiologist. (B), ICC(2,1) with 95% bootstrap confidence intervals for ROI-derived tumor volume and maximum axial diameter. (C), ICC distribution for all 1,326 nonconstant PyRadiomics features with valid estimates. Dashed and dotted lines indicate ICC thresholds of 0.75 and 0.90, respectively.

**Supplementary Figure 2. Single-modality and fused phenotype analyses** (A-D), Kaplan-Meier curves showing DFS and OS separation for CRCFound-only and PyRadiomics-only clusters in the SYSU6H cohort 2. (E), Silhouette comparison for CRCFound-only, PyRadiomics-only, and fused multimodal representations at k = 2. (F), Sensitivity analysis of silhouette coefficients across candidate cluster numbers for the fused representation. These panels support the complementary roles of CRCFound and PyRadiomics in imaging phenotype derivation and provide additional evidence for the selected k = 2 fused solution.

**Supplementary Figure 3. Sensitivity analysis with and without Cox-based feature screening** (A), Harrell C-index for DFS comparing the principal CT phenotypes with the k = 2 sensitivity solution without the modality-specific univariable Cox feature-screening step. (B), Corresponding Harrell C-index for OS. (C), Number of Hallmark pathways differing between groups at BH-FDR < 0.05. The two x-axis categories indicate whether Cox feature screening was included before dimensionality reduction and clustering. Because binary cluster labels are arbitrary, C-indices for the sensitivity solution were oriented to the prognostic direction using max(C, 1-C).

**Supplementary Figure 4. Tumor size comparisons and Cox sensitivity analyses** (A,B), ROI-derived tumor volume (A) and maximum axial diameter (B) by CT phenotype in SYSU6H cohort 2 and Ningbo cohort 1. P values are from two-sided Mann-Whitney U tests. (C,D), Complete-case multivariable Cox forest plots for DFS (C) and OS (D) in SYSU6H cohort 2 without tumor-size covariates. These models included CT phenotype, TNM stage, MSI status, tumor side, grade, BMI, sex, and age. Figure 3A,B show the principal models including tumor-size covariates, whereas Supplementary Figure 4C,D show the corresponding models without tumor-size covariates; the estimates differed little. Paired estimates for SYSU6H cohort 2 and Ningbo cohort 1 are reported in Supplementary Table 5. P values for Cox-model coefficients are from two-sided Wald tests. * P < 0.05; ** P < 0.01; *** P < 0.001. No adjustment for multiple comparisons was applied to these coefficient-level P values.

**Supplementary Figure 5. Stage-stratified clinical and survival analyses of CT phenotypes** (A-D), Kaplan-Meier curves for DFS by CT phenotype within TNM stages I-IV. (E-H), Kaplan-Meier curves for OS by CT phenotype within TNM stages I-IV. HRs are from Cox models and P values are from log-rank tests. (I), Proportion of CT-S1 and CT-S2 across TNM stages I-IV in the SYSU6H cohort 2. These analyses evaluate whether phenotype-associated survival separation is retained within anatomical stage strata.

**Supplementary Figure 6. Additional molecular and pathway correlates of CT phenotypes** (A), The 11 of 50 HALLMARK pathways with BH-FDR < 0.05, shown as differences between CT-S2 and CT-S1. Positive values indicate higher pathway scores in CT-S2, and negative values indicate higher pathway scores in CT-S1. (B), Median expression differences in selected immune and microenvironment-related markers between CT-S2 and CT-S1. Bar color indicates the CT phenotype with higher values, and circle size denotes -log10(P). These analyses provide supportive transcriptomic context for the pathway and immune-marker differences associated with the CT phenotypes.

**Supplementary Figure 7. External 3-year model evaluation in Ningbo cohort 1** (A,B), Calibration of 3-year DFS (A) and OS (B) event probabilities in complete cases from Ningbo cohort 1 (DFS, n = 400; OS, n = 404). Observed probabilities are Kaplan-Meier estimates within prediction strata. (C,D), IPCW decision curves for 3-year DFS (C) and OS (D). Curves compare a clinical base Cox model including age, sex, grade, TNM stage, tumor side, and MSI status with the same model plus the locked CT phenotype. Models were fitted in SYSU6H cohort 2 and evaluated without refitting in Ningbo cohort 1. The dashed diagonal in A and B denotes ideal calibration; decision curves include the Treat all and Treat none reference strategies. The estimated probabilities of remaining uncensored at 3 years were 0.318 for DFS and 0.348 for OS, so external estimates should be interpreted in the context of limited follow-up support.

**Supplementary Figure 8. Internal cross-validated 3-year model evaluation in SYSU6H cohort 2** (A,B), Five-fold cross-validated calibration of 3-year DFS (A) and OS (B) event probabilities in complete cases from SYSU6H cohort 2 (n = 2,657 for each endpoint). Observed probabilities are Kaplan-Meier estimates within prediction strata. (C,D), IPCW decision curves for 3-year DFS (C) and OS (D). Curves compare a clinical base Cox model including age, sex, grade, TNM stage, tumor side, and MSI status with the same model plus the locked CT phenotype. Age standardization, Cox-model fitting, and baseline-risk estimation were performed within each training fold; the locked phenotype labels were not re-derived within folds. The dashed diagonal in A and B denotes ideal calibration; decision curves include the Treat all and Treat none reference strategies. This is a conditional cross-validated evaluation of the Cox layer using fixed phenotype labels, not an end-to-end cross-validation of phenotype derivation.

**Supplementary Figure 9. Baseline clinical feature distributions in SYSU6HNAT** (A-D), Within-cluster distributions of grade (A), CEA category (B; ng/mL), CA19-9 category (C; U/mL), and MMR status (D) in the SYSU6HNAT neoadjuvant-treatment cohort. P values are from chi-square tests or Fisher’s exact tests as appropriate.
