# Supplementary material for: Integrated CT Foundation Model and Radiomic Features Define Clinically and Biologically Distinct Colorectal Cancer Subtypes
Source: Research (Wash D C). 2026 Oct 6;9:1435. doi: 10.34133/research.1435 (PMC13639147; doi:10.34133/research.1435)
Supplement: Supplementary file 1 [file research.1435.f1.zip › Tables S1 to S7.docx]

**Supplementary Tables**

This file contains 16 supplementary table panels.

Supplementary Table 1. Clinical characteristics by cohort and, where applicable, CT subtype

Supplementary Table 2A. Cohort roles and partition basis

Supplementary Table 2B. Center-specific CT scanner and acquisition characteristics

Supplementary Table 3A. Interobserver spatial agreement in random 30 cases

Supplementary Table 3B. ICC(2,1) for ROI-derived size

Supplementary Table 3C. PyRadiomics ICC summary

Supplementary Table 4. Sensitivity analysis without Cox-based feature screening

Supplementary Table 5A. ROI-derived tumor size by CT phenotype

Supplementary Table 5B. Two-sided comparisons of tumor size between CT phenotypes

Supplementary Table 5C. Multivariable Cox models in Ningbo cohort 1 with tumor-size adjustment

Supplementary Table 5D. Paired Cox sensitivity analyses with and without tumor-size adjustment

Supplementary Table 6A. Baseline characteristics by Transcriptome/CMS data availability within SYSU6H cohort 2

Supplementary Table 6B. Baseline characteristics by Mutation data availability within SYSU6H cohort 2

Supplementary Table 6C. Baseline characteristics by Ki-67 data availability within SYSU6H cohort 2

Supplementary Table 7A. IPCW 3-year performance of clinical models

Supplementary Table 7B. Incremental performance and IPCW continuous NRI/IDI after adding CT phenotype

**Supplementary Table 1. Clinical characteristics by cohort and, where applicable, CT subtype**

| **Cohort** | **Variable** | **Level** | **Overall (n (%) for categorical variables)** | **CT-S1** | **CT-S2** | **P value** | **Test** | **Non-missing N** | **Source variable** |
| --- | --- | --- | --- | --- | --- | --- | --- | --- | --- |
| SYSU6H cohort 1 (fine-tuning) | n |  | 902 |  |  |  |  |  |  |
| SYSU6H cohort 1 (fine-tuning) | Age, years, mean (SD) |  | 58.65 (13.84) |  |  |  |  | 902 | age |
| SYSU6H cohort 1 (fine-tuning) | BMI, kg/m^2, mean (SD) |  | 22.44 (3.43) |  |  |  |  | 894 | BMI |
| SYSU6H cohort 1 (fine-tuning) | DFS time, months, mean (SD) |  | 49.92 (29.24) |  |  |  |  | 902 | dfs_time/dfs.delay |
| SYSU6H cohort 1 (fine-tuning) | DFS time, months, median (IQR) |  | 61.47 (18.41-69.38) |  |  |  |  | 902 | dfs_time/dfs.delay |
| SYSU6H cohort 1 (fine-tuning) | OS time, months, mean (SD) |  | 54.51 (26.63) |  |  |  |  | 902 | os_time/os.delay |
| SYSU6H cohort 1 (fine-tuning) | OS time, months, median (IQR) |  | 62.72 (31.54-71.15) |  |  |  |  | 902 | os_time/os.delay |
| SYSU6H cohort 1 (fine-tuning) | Sex | Female | 360 (39.9) |  |  |  |  | 902 | sex |
| SYSU6H cohort 1 (fine-tuning) |  | Male | 542 (60.1) |  |  |  |  |  |  |
| SYSU6H cohort 1 (fine-tuning) | Tumor side | Left-sided | 467 (60.5) |  |  |  |  | 772 | tumor.location |
| SYSU6H cohort 1 (fine-tuning) |  | Right-sided | 305 (39.5) |  |  |  |  |  |  |
| SYSU6H cohort 1 (fine-tuning) | Grade | Grade 1 | 110 (13.6) |  |  |  |  | 806 | grade |
| SYSU6H cohort 1 (fine-tuning) |  | Grade 2 | 647 (80.3) |  |  |  |  |  |  |
| SYSU6H cohort 1 (fine-tuning) |  | Grade 3 | 42 (5.2) |  |  |  |  |  |  |
| SYSU6H cohort 1 (fine-tuning) |  | Grade 4 | 7 (0.9) |  |  |  |  |  |  |
| SYSU6H cohort 1 (fine-tuning) | TNM stage | Stage I | 69 (7.8) |  |  |  |  | 885 | tnm.stage |
| SYSU6H cohort 1 (fine-tuning) |  | Stage II | 377 (42.6) |  |  |  |  |  |  |
| SYSU6H cohort 1 (fine-tuning) |  | Stage III | 264 (29.8) |  |  |  |  |  |  |
| SYSU6H cohort 1 (fine-tuning) |  | Stage IV | 175 (19.8) |  |  |  |  |  |  |
| SYSU6H cohort 1 (fine-tuning) | MSI/MMR status | MSS | 695 (79.1) |  |  |  |  | 879 | mmr.status |
| SYSU6H cohort 1 (fine-tuning) |  | MSI-H | 184 (20.9) |  |  |  |  |  |  |
| SYSU6H cohort 1 (fine-tuning) | DFS event | No event | 564 (62.5) |  |  |  |  | 902 | dfs_event/dfs.event |
| SYSU6H cohort 1 (fine-tuning) |  | Event | 338 (37.5) |  |  |  |  |  |  |
| SYSU6H cohort 1 (fine-tuning) | OS event | No event | 619 (68.6) |  |  |  |  | 902 | os_event/os.event |
| SYSU6H cohort 1 (fine-tuning) |  | Event | 283 (31.4) |  |  |  |  |  |  |
| SYSU6H cohort 2 (discovery) | n |  | 3267 | 1835 | 1432 |  |  |  | Subtype |
| SYSU6H cohort 2 (discovery) | Age, years, mean (SD) |  | 58.59 (12.70) | 57.67 (12.39) | 59.77 (12.99) | <0.001 | Wilcoxon rank-sum | 3072 | age |
| SYSU6H cohort 2 (discovery) | BMI, kg/m^2, mean (SD) |  | 22.84 (3.23) | 23.08 (3.15) | 22.52 (3.29) | <0.001 | Wilcoxon rank-sum | 3218 | BMI |
| SYSU6H cohort 2 (discovery) | DFS time, months, mean (SD) |  | 47.33 (27.57) | 56.52 (26.30) | 35.56 (24.52) | <0.001 | Wilcoxon rank-sum | 3267 | dfs_time/dfs.delay |
| SYSU6H cohort 2 (discovery) | DFS time, months, median (IQR) |  | 43.36 (28.66-61.27) | 51.35 (36.69-71.47) | 31.91 (16.48-48.38) |  |  | 3267 | dfs_time/dfs.delay |
| SYSU6H cohort 2 (discovery) | OS time, months, mean (SD) |  | 52.14 (32.97) | 58.58 (37.95) | 43.89 (22.69) | <0.001 | Wilcoxon rank-sum | 3267 | os_time/os.delay |
| SYSU6H cohort 2 (discovery) | OS time, months, median (IQR) |  | 46.62 (33.52-64.22) | 52.41 (38.56-72.86) | 39.78 (28.87-55.46) |  |  | 3267 | os_time/os.delay |
| SYSU6H cohort 2 (discovery) | Sex | Female | 1325 (40.6) | 804 (43.8) | 521 (36.4) | <0.001 | Fisher exact/chi-square | 3267 | sex |
| SYSU6H cohort 2 (discovery) |  | Male | 1942 (59.4) | 1031 (56.2) | 911 (63.6) |  |  |  |  |
| SYSU6H cohort 2 (discovery) | Tumor side | Left-sided | 2503 (76.8) | 1363 (74.4) | 1140 (79.8) | <0.001 | Fisher exact/chi-square | 3261 | tumor.location |
| SYSU6H cohort 2 (discovery) |  | Right-sided | 758 (23.2) | 470 (25.6) | 288 (20.2) |  |  |  |  |
| SYSU6H cohort 2 (discovery) | Grade | Grade 1 | 526 (18.5) | 318 (20.0) | 208 (16.7) | 0.005 | Fisher exact/chi-square | 2836 | grade |
| SYSU6H cohort 2 (discovery) |  | Grade 2 | 2192 (77.3) | 1224 (76.8) | 968 (77.9) |  |  |  |  |
| SYSU6H cohort 2 (discovery) |  | Grade 3 | 97 (3.4) | 40 (2.5) | 57 (4.6) |  |  |  |  |
| SYSU6H cohort 2 (discovery) |  | Grade 4 | 21 (0.7) | 11 (0.7) | 10 (0.8) |  |  |  |  |
| SYSU6H cohort 2 (discovery) | TNM stage | Stage I | 534 (16.4) | 368 (20.2) | 166 (11.6) | <0.001 | Fisher exact/chi-square | 3251 | tnm.stage |
| SYSU6H cohort 2 (discovery) |  | Stage II | 1301 (40.0) | 802 (44.0) | 499 (35.0) |  |  |  |  |
| SYSU6H cohort 2 (discovery) |  | Stage III | 1141 (35.1) | 607 (33.3) | 534 (37.4) |  |  |  |  |
| SYSU6H cohort 2 (discovery) |  | Stage IV | 275 (8.5) | 47 (2.6) | 228 (16.0) |  |  |  |  |
| SYSU6H cohort 2 (discovery) | MSI/MMR status | MSS | 2819 (92.1) | 1558 (90.1) | 1261 (94.7) | <0.001 | Fisher exact/chi-square | 3061 | mmr.status |
| SYSU6H cohort 2 (discovery) |  | MSI-H | 242 (7.9) | 171 (9.9) | 71 (5.3) |  |  |  |  |
| SYSU6H cohort 2 (discovery) | DFS event | No event | 2437 (74.6) | 1714 (93.4) | 723 (50.5) | <0.001 | Fisher exact/chi-square | 3267 | dfs_event/dfs.event |
| SYSU6H cohort 2 (discovery) |  | Event | 830 (25.4) | 121 (6.6) | 709 (49.5) |  |  |  |  |
| SYSU6H cohort 2 (discovery) | OS event | No event | 2755 (84.3) | 1778 (96.9) | 977 (68.2) | <0.001 | Fisher exact/chi-square | 3267 | os_event/os.event |
| SYSU6H cohort 2 (discovery) |  | Event | 512 (15.7) | 57 (3.1) | 455 (31.8) |  |  |  |  |
| Ningbo cohort 1 (external validation) | n |  | 509 | 256 | 253 |  |  |  | Subtype |
| Ningbo cohort 1 (external validation) | Age, years, mean (SD) |  | 65.57 (10.51) | 64.88 (10.04) | 66.26 (10.93) | 0.115 | Wilcoxon rank-sum | 486 | age |
| Ningbo cohort 1 (external validation) | BMI, kg/m^2, mean (SD) |  | 23.19 (3.10) | 23.42 (3.09) | 22.96 (3.11) | 0.172 | Wilcoxon rank-sum | 445 | BMI |
| Ningbo cohort 1 (external validation) | DFS time, months, mean (SD) |  | 25.65 (12.79) | 29.45 (11.49) | 21.63 (12.90) | <0.001 | Wilcoxon rank-sum | 494 | dfs_time/dfs.delay |
| Ningbo cohort 1 (external validation) | DFS time, months, median (IQR) |  | 25.43 (18.79-36.25) | 30.17 (23.55-37.72) | 23.05 (11.07-31.87) |  |  | 494 | dfs_time/dfs.delay |
| Ningbo cohort 1 (external validation) | OS time, months, mean (SD) |  | 28.03 (12.14) | 30.25 (11.36) | 25.80 (12.50) | <0.001 | Wilcoxon rank-sum | 502 | os_time/os.delay |
| Ningbo cohort 1 (external validation) | OS time, months, median (IQR) |  | 27.87 (22.47-37.34) | 31.75 (24.05-38.27) | 25.63 (19.14-35.64) |  |  | 502 | os_time/os.delay |
| Ningbo cohort 1 (external validation) | Sex | Female | 188 (38.7) | 102 (42.0) | 86 (35.4) | 0.162 | Fisher exact/chi-square | 486 | sex |
| Ningbo cohort 1 (external validation) |  | Male | 298 (61.3) | 141 (58.0) | 157 (64.6) |  |  |  |  |
| Ningbo cohort 1 (external validation) | Tumor side | Left-sided | 347 (78.2) | 168 (76.4) | 179 (79.9) | 0.422 | Fisher exact/chi-square | 444 | tumor.location |
| Ningbo cohort 1 (external validation) |  | Right-sided | 97 (21.8) | 52 (23.6) | 45 (20.1) |  |  |  |  |
| Ningbo cohort 1 (external validation) | Grade | Grade 1 | 13 (2.9) | 4 (1.8) | 9 (3.9) | 0.052 | Fisher exact/chi-square | 454 | grade |
| Ningbo cohort 1 (external validation) |  | Grade 2 | 296 (65.2) | 157 (70.4) | 139 (60.2) |  |  |  |  |
| Ningbo cohort 1 (external validation) |  | Grade 3 | 145 (31.9) | 62 (27.8) | 83 (35.9) |  |  |  |  |
| Ningbo cohort 1 (external validation) | TNM stage | Stage I | 82 (17.8) | 42 (18.3) | 40 (17.2) | <0.001 | Fisher exact/chi-square | 461 | tnm.stage |
| Ningbo cohort 1 (external validation) |  | Stage II | 156 (33.8) | 91 (39.7) | 65 (28.0) |  |  |  |  |
| Ningbo cohort 1 (external validation) |  | Stage III | 169 (36.7) | 83 (36.2) | 86 (37.1) |  |  |  |  |
| Ningbo cohort 1 (external validation) |  | Stage IV | 54 (11.7) | 13 (5.7) | 41 (17.7) |  |  |  |  |
| Ningbo cohort 1 (external validation) | MSI/MMR status | MSS | 422 (94.4) | 204 (93.2) | 218 (95.6) | 0.306 | Fisher exact/chi-square | 447 | mmr.status |
| Ningbo cohort 1 (external validation) |  | MSI-H | 25 (5.6) | 15 (6.8) | 10 (4.4) |  |  |  |  |
| Ningbo cohort 1 (external validation) | DFS event | No event | 424 (83.3) | 250 (97.7) | 174 (68.8) | <0.001 | Fisher exact/chi-square | 509 | dfs_event/dfs.event |
| Ningbo cohort 1 (external validation) |  | Event | 85 (16.7) | 6 (2.3) | 79 (31.2) |  |  |  |  |
| Ningbo cohort 1 (external validation) | OS event | No event | 458 (90.0) | 253 (98.8) | 205 (81.0) | <0.001 | Fisher exact/chi-square | 509 | os_event/os.event |
| Ningbo cohort 1 (external validation) |  | Event | 51 (10.0) | 3 (1.2) | 48 (19.0) |  |  |  |  |
| SYSU6HNAT cohort (neoadjuvant) | n |  | 709 | 260 | 449 |  |  |  | Subtype |
| SYSU6HNAT cohort (neoadjuvant) | Age, years, mean (SD) |  | 58.34 (11.54) | 58.87 (11.24) | 58.03 (11.71) | 0.369 | Wilcoxon rank-sum | 709 | age |
| SYSU6HNAT cohort (neoadjuvant) | DFS time, months, mean (SD) |  | 38.36 (28.92) | 55.13 (31.18) | 28.64 (22.41) | <0.001 | Wilcoxon rank-sum | 709 | dfs_time/dfs.delay |
| SYSU6HNAT cohort (neoadjuvant) | DFS time, months, median (IQR) |  | 31.84 (16.15-51.71) | 47.19 (30.21-72.42) | 22.80 (12.12-38.61) |  |  | 709 | dfs_time/dfs.delay |
| SYSU6HNAT cohort (neoadjuvant) | OS time, months, mean (SD) |  | 44.42 (27.66) | 56.10 (31.19) | 37.65 (22.83) | <0.001 | Wilcoxon rank-sum | 709 | os_time/os.delay |
| SYSU6HNAT cohort (neoadjuvant) | OS time, months, median (IQR) |  | 38.14 (24.10-58.91) | 47.70 (30.66-74.76) | 34.72 (20.27-48.79) |  |  | 709 | os_time/os.delay |
| SYSU6HNAT cohort (neoadjuvant) | Sex | Female | 199 (28.1) | 74 (28.5) | 125 (27.8) | 0.863 | Fisher exact/chi-square | 709 | sex |
| SYSU6HNAT cohort (neoadjuvant) |  | Male | 510 (71.9) | 186 (71.5) | 324 (72.2) |  |  |  |  |
| SYSU6HNAT cohort (neoadjuvant) | Tumor side | Left-sided | 698 (100.0) | 256 (100.0) | 442 (100.0) |  | Fisher exact/chi-square | 698 | tumor.location |
| SYSU6HNAT cohort (neoadjuvant) | Grade | Grade 1 | 58 (13.4) | 28 (17.1) | 30 (11.1) | 0.089 | Fisher exact/chi-square | 434 | grade |
| SYSU6HNAT cohort (neoadjuvant) |  | Grade 2 | 335 (77.2) | 125 (76.2) | 210 (77.8) |  |  |  |  |
| SYSU6HNAT cohort (neoadjuvant) |  | Grade 3 | 41 (9.4) | 11 (6.7) | 30 (11.1) |  |  |  |  |
| SYSU6HNAT cohort (neoadjuvant) | Clinical TNM stage | Stage II | 256 (36.1) | 97 (37.3) | 159 (35.4) | 0.627 | Fisher exact/chi-square | 709 | cTNM |
| SYSU6HNAT cohort (neoadjuvant) |  | Stage III | 453 (63.9) | 163 (62.7) | 290 (64.6) |  |  |  |  |
| SYSU6HNAT cohort (neoadjuvant) |  | Stage IV | 0 (0.0) | 0 (0.0) | 0 (0.0) |  |  |  |  |
| SYSU6HNAT cohort (neoadjuvant) | MSI/MMR status | MSS | 508 (98.1) | 162 (97.0) | 346 (98.6) | 0.304 | Fisher exact/chi-square | 518 | mmr.status |
| SYSU6HNAT cohort (neoadjuvant) |  | MSI-H | 10 (1.9) | 5 (3.0) | 5 (1.4) |  |  |  |  |
| SYSU6HNAT cohort (neoadjuvant) | DFS event | No event | 494 (69.7) | 252 (96.9) | 242 (53.9) | <0.001 | Fisher exact/chi-square | 709 | dfs_event/dfs.event |
| SYSU6HNAT cohort (neoadjuvant) |  | Event | 215 (30.3) | 8 (3.1) | 207 (46.1) |  |  |  |  |
| SYSU6HNAT cohort (neoadjuvant) | OS event | No event | 602 (84.9) | 257 (98.8) | 345 (76.8) | <0.001 | Fisher exact/chi-square | 709 | os_event/os.event |
| SYSU6HNAT cohort (neoadjuvant) |  | Event | 107 (15.1) | 3 (1.2) | 104 (23.2) |  |  |  |  |

*Note: SYSU6H cohort 1 is shown overall only because it was used exclusively for CRCFound fine-tuning and was not assigned CT subtypes for the study analyses. Abbreviations: BMI, body mass index; cTNM, clinical tumor-node-metastasis stage; CT, computed tomography; DFS, disease-free survival; IQR, interquartile range; MMR, mismatch repair; MSI, microsatellite instability; MSI-H, microsatellite instability-high; MSS, microsatellite stable; OS, overall survival; SD, standard deviation; SYSU6H, Sixth Affiliated Hospital of Sun Yat-sen University; SYSU6HNAT, SYSU6H neoadjuvant-treatment cohort; TNM, tumor-node-metastasis.*

**Supplementary Table 2A. Cohort roles and partition basis**

| **Cohort** | **N** | **Role** | **Partition basis** | **Real manual ROI** | **Phenotype derivation** | **External evaluation** |
| --- | --- | --- | --- | --- | --- | --- |
| SYSU6H cohort 1 | 902 | CRCFound fine-tuning | Independent CT-focused project | Yes | No | No |
| SYSU6H cohort 2 | 3267 | Phenotype derivation and linked analyses | Independent multimodal CRC project | Yes | Yes | No |
| Ningbo cohort 1 | 509 | External evaluation | Independent institution | Yes | No | Yes |
| SYSU6HNAT | 709 | Neoadjuvant evaluation | Neoadjuvant treatment-defined | No; geometrically centered CRCFound-only input; no PyRadiomics or ROI-derived size | No | No |

*Abbreviations: CRC, colorectal cancer; CT, computed tomography; ROI, region of interest; SYSU6H, Sixth Affiliated Hospital of Sun Yat-sen University; SYSU6HNAT, SYSU6H neoadjuvant-treatment cohort.*

**Supplementary Table 2B. Center-specific CT scanner and acquisition characteristics**

| **Center** | **Applicable cohorts** | **Scanner systems, manufacturer and location, %** | **Contrast-enhanced phase** | **Slice thickness, mm, median (IQR); range** | **Tube voltage, kVp, %** | **Tube current, mA, median (IQR); range** | **Reconstruction kernel, %** |
| --- | --- | --- | --- | --- | --- | --- | --- |
| SYSU6H | SYSU6H cohort 1 (N = 902); SYSU6H cohort 2 (N = 3267); SYSU6HNAT (N = 709) | 64-detector-row Optima CT660 (GE Healthcare, Milwaukee, WI, USA): 52.0% 320-detector-row Aquilion ONE (Toshiba Medical Systems, Otawara, Japan): 47.7% 64-detector-row/128-slice uCT 760 (United Imaging Healthcare, Shanghai, China): 0.3% | Venous-phase contrast-enhanced CT | 3.8 (3.0-3.8); range 0.8-7.5 | 120: 99.7%; 100: 0.3% | 326 (269-364); range 50-550 | STANDARD: 52.0%; FC08: 38.5%; FL03: 5.4%; FC19: 3.8%; B_SOFT_D: 0.2%; B_SOFT_C: 0.1% |
| Ningbo | Ningbo cohort 1 (N = 509) | 16-detector-row Brilliance 16 (Philips Healthcare, Cleveland, OH, USA): 86.7% 256-detector-row Revolution CT (GE Healthcare, Milwaukee, WI, USA): 7.3% 64-detector-row/128-slice Incisive CT (Philips Healthcare, Cleveland, OH, USA): 4.2% Ingenuity Flex (Philips Healthcare, Cleveland, OH, USA): 0.8% 16-detector-row MX 16-slice (Philips Healthcare, Cleveland, OH, USA): 0.6% 320-detector-row/640-slice uCT 960+ (United Imaging Healthcare, Shanghai, China): 0.3% | Venous-phase contrast-enhanced CT | 2.0 (2.0-2.0); range 0.6-5.0 | 120: 98.6%; 100: 1.4% | 240 (215-263); range 88-479 | B: 91.8%; STANDARD: 7.3%; SB: 0.6%; B_SOFT_A: 0.3% |

*Abbreviations: CT, computed tomography; IQR, interquartile range; kVp, peak kilovoltage; mA, milliampere; OH, Ohio; SYSU6H, Sixth Affiliated Hospital of Sun Yat-sen University; SYSU6HNAT, SYSU6H neoadjuvant-treatment cohort; USA, United States of America; WI, Wisconsin.*

**Supplementary Table 3A. Interobserver spatial agreement in random 30 cases**

| **metric** | **n** | **mean** | **sd** | **median** | **q1** | **q3** | **min** | **max** |
| --- | --- | --- | --- | --- | --- | --- | --- | --- |
| dice | 30 | 0.924 | 0.053 | 0.933 | 0.892 | 0.972 | 0.808 | 0.988 |
| jaccard | 30 | 0.863 | 0.089 | 0.874 | 0.805 | 0.945 | 0.678 | 0.977 |
| hd95_mm | 30 | 3.780 | 4.500 | 2.236 | 1 | 4.083 | 0 | 17.493 |
| assd_mm | 30 | 0.688 | 0.938 | 0.325 | 0.129 | 0.681 | 0.034 | 4.123 |
| centroid_distance_mm | 30 | 1.749 | 1.992 | 1.100 | 0.397 | 1.933 | 0.034 | 8.019 |

*Abbreviations: ASSD, average symmetric surface distance; HD95, 95th-percentile Hausdorff distance; Q1, first quartile; Q3, third quartile; SD, standard deviation.*

**Supplementary Table 3B. ICC(2,1) for ROI-derived size**

| **metric** | **icc_model** | **n** | **icc** | **bootstrap_ci95_lower** | **bootstrap_ci95_upper** |
| --- | --- | --- | --- | --- | --- |
| tumor_volume_cm3 | ICC(2,1), two-way random-effects, absolute agreement, single measurement | 30 | 0.977 | 0.951 | 0.990 |
| maximum_axial_diameter_mm | ICC(2,1), two-way random-effects, absolute agreement, single measurement | 30 | 0.985 | 0.953 | 0.999 |

*Abbreviations: CI, confidence interval; ICC, intraclass correlation coefficient; ROI, region of interest.*

**Supplementary Table 3C. PyRadiomics ICC summary**

| **scope** | **n_valid_features** | **median_icc** | **q1_icc** | **q3_icc** | **n_icc_ge_0_75** | **percent_icc_ge_0_75** | **n_icc_ge_0_90** | **percent_icc_ge_0_90** |
| --- | --- | --- | --- | --- | --- | --- | --- | --- |
| all_features | 1326 | 0.972 | 0.943 | 0.987 | 1326 | 100 | 1231 | 92.8 |

*Abbreviations: ICC, intraclass correlation coefficient; Q1, first quartile; Q3, third quartile.*

**Supplementary Table 4. Sensitivity analysis without Cox-based feature screening**

| **Clustering scheme** | **k** | **N** | **DFS Harrell C-index** | **OS Harrell C-index** | **DFS log-rank P** | **OS log-rank P** | **Hallmark pathways at BH-FDR < 0.05** | **Total Hallmark pathways** |
| --- | --- | --- | --- | --- | --- | --- | --- | --- |
| Without Cox feature screening | 2 | 3267 | 0.501 | 0.506 | 0.763 | 0.662 | 2 | 50 |
| Principal CT phenotype | 2 | 3267 | 0.756 | 0.769 | <0.0001 | <0.0001 | 11 | 50 |

*Abbreviations: BH-FDR, Benjamini-Hochberg false discovery rate; C-index, concordance index; CT, computed tomography; DFS, disease-free survival; OS, overall survival.*

**Supplementary Table 5A. ROI-derived tumor size by CT phenotype**

| **Cohort** | **CT phenotype** | **N** | **Tumor volume (cm^3), median (Q1-Q3)** | **Maximum axial diameter (mm), median (Q1-Q3)** |
| --- | --- | --- | --- | --- |
| Ningbo cohort 1 | CT-S1 | 256 | 24.33 (15.04-44.61) | 44.24 (36.86-55.09) |
| Ningbo cohort 1 | CT-S2 | 253 | 30.22 (18.01-50.05) | 47.71 (40.22-56.08) |
| SYSU6H cohort 2 | CT-S1 | 1835 | 18.53 (8.82-36.39) | 54.74 (42.72-69.71) |
| SYSU6H cohort 2 | CT-S2 | 1432 | 25.75 (12.73-48.57) | 57.76 (46.37-77.49) |

*Values are median (Q1-Q3), where Q1 and Q3 denote the 25th and 75th percentiles, respectively. Tumor volume is reported in cm^3 and maximum axial diameter in mm. Abbreviations: CT, computed tomography; ROI, region of interest; SYSU6H, Sixth Affiliated Hospital of Sun Yat-sen University.*

**Supplementary Table 5B. Two-sided comparisons of tumor size between CT phenotypes**

| **Cohort** | **Measure** | **N** | **P value** |
| --- | --- | --- | --- |
| Ningbo cohort 1 | Tumor volume (cm^3) | 509 | 0.006 |
| Ningbo cohort 1 | Maximum axial diameter (mm) | 509 | 0.011 |
| SYSU6H cohort 2 | Tumor volume (cm^3) | 3267 | <0.001 |
| SYSU6H cohort 2 | Maximum axial diameter (mm) | 3267 | <0.001 |

*P values are from two-sided Mann-Whitney U tests comparing CT-S1 and CT-S2 within each cohort. Abbreviations: CT, computed tomography; SYSU6H, Sixth Affiliated Hospital of Sun Yat-sen University.*

**Supplementary Table 5C. Multivariable Cox models in Ningbo cohort 1 with tumor-size adjustment**

| **Endpoint** | **Cohort** | **N** | **Events** | **Variable** | **HR** | **95% CI** | **P value** |
| --- | --- | --- | --- | --- | --- | --- | --- |
| DFS | Ningbo cohort 1 | 450 | 77 | CT-S2 vs CT-S1 | 13.92 | 6.01-32.20 | <0.001 |
| DFS | Ningbo cohort 1 | 450 | 77 | Age (per year) | 1.00 | 0.98-1.02 | 0.983 |
| DFS | Ningbo cohort 1 | 450 | 77 | Male vs female | 1.08 | 0.67-1.73 | 0.749 |
| DFS | Ningbo cohort 1 | 450 | 77 | TNM stage III-IV vs I-II | 4.55 | 2.60-7.97 | <0.001 |
| DFS | Ningbo cohort 1 | 450 | 77 | Maximum axial diameter (per 10 mm) | 0.83 | 0.67-1.03 | 0.095 |
| DFS | Ningbo cohort 1 | 450 | 77 | Tumor volume (per doubling) | 1.57 | 1.14-2.16 | 0.005 |
| OS | Ningbo cohort 1 | 455 | 44 | CT-S2 vs CT-S1 | 20.24 | 4.86-84.26 | <0.001 |
| OS | Ningbo cohort 1 | 455 | 44 | Age (per year) | 1.04 | 1.01-1.07 | 0.022 |
| OS | Ningbo cohort 1 | 455 | 44 | Male vs female | 0.85 | 0.46-1.58 | 0.610 |
| OS | Ningbo cohort 1 | 455 | 44 | TNM stage III-IV vs I-II | 3.14 | 1.57-6.28 | 0.001 |
| OS | Ningbo cohort 1 | 455 | 44 | Maximum axial diameter (per 10 mm) | 0.86 | 0.64-1.16 | 0.339 |
| OS | Ningbo cohort 1 | 455 | 44 | Tumor volume (per doubling) | 1.30 | 0.87-1.92 | 0.200 |

*Unpenalized complete-case Cox proportional-hazards models included CT phenotype, age, sex, grouped TNM stage, maximum axial diameter per 10-mm increase, and log2-transformed tumor volume per doubling; a one-unit increase in log2-transformed tumor volume corresponds to a doubling of tumor volume. HR compares CT-S2 with CT-S1. Abbreviations: CI, confidence interval; CT, computed tomography; DFS, disease-free survival; HR, hazard ratio; OS, overall survival; TNM, tumor-node-metastasis.*

**Supplementary Table 5D. Paired Cox sensitivity analyses with and without tumor-size adjustment**

| **Cohort** | **Endpoint** | **Model** | **N** | **Events** | **CT-S2 HR** | **95% CI** | **P value** |
| --- | --- | --- | --- | --- | --- | --- | --- |
| SYSU6H cohort 2 | DFS | Clinical + CT phenotype | 2533 | 591 | 8.85 | 6.97-11.24 | <0.001 |
| SYSU6H cohort 2 | DFS | Clinical + CT phenotype + tumor size | 2533 | 591 | 8.80 | 6.93-11.19 | <0.001 |
| SYSU6H cohort 2 | OS | Clinical + CT phenotype | 2533 | 325 | 11.32 | 7.75-16.55 | <0.001 |
| SYSU6H cohort 2 | OS | Clinical + CT phenotype + tumor size | 2533 | 325 | 10.91 | 7.46-15.96 | <0.001 |
| Ningbo cohort 1 | DFS | Clinical + CT phenotype | 450 | 77 | 15.04 | 6.51-34.71 | <0.001 |
| Ningbo cohort 1 | DFS | Clinical + CT phenotype + tumor size | 450 | 77 | 13.92 | 6.01-32.20 | <0.001 |
| Ningbo cohort 1 | OS | Clinical + CT phenotype | 455 | 44 | 21.09 | 5.08-87.53 | <0.001 |
| Ningbo cohort 1 | OS | Clinical + CT phenotype + tumor size | 455 | 44 | 20.24 | 4.86-84.26 | <0.001 |
|  |  |  |  |  |  |  |  |

*Each endpoint used an identical complete-case set for the paired models. Tumor-size-adjusted models additionally included maximum axial diameter per 10-mm increase and log2-transformed tumor volume per doubling. All models were ordinary unpenalized Cox proportional-hazards models. Abbreviations: CI, confidence interval; CT, computed tomography; DFS, disease-free survival; HR, hazard ratio; OS, overall survival; SYSU6H, Sixth Affiliated Hospital of Sun Yat-sen University.*

**Supplementary Table 6A. Baseline characteristics by Transcriptome/CMS data availability within SYSU6H cohort 2 (available n = 481; unavailable n = 2786)**

| **Variable** | **Level** | **Available non-missing N** | **Available, median (IQR) or n (%)** | **Unavailable non-missing N** | **Unavailable, median (IQR) or n (%)** | **Test** | **P value** | **BH-FDR** | **Absolute SMD** |
| --- | --- | --- | --- | --- | --- | --- | --- | --- | --- |
| Age, years |  | 355 | 58.00 (48.00-67.50) | 2717 | 60.00 (51.00-68.00) | Mann-Whitney U test | 0.04 | 0.053 | 0.122 |
| BMI, kg/m^2 |  | 477 | 22.49 (20.20-24.44) | 2741 | 22.76 (20.76-24.85) | Mann-Whitney U test | 0.006 | 0.010 | 0.164 |
| Tumor volume, cm^3 |  | 481 | 27.58 (14.33-51.36) | 2786 | 20.00 (9.58-40.47) | Mann-Whitney U test | <0.001 | <0.001 | 0.025 |
| Maximum axial diameter, mm |  | 481 | 51.35 (42.15-65.92) | 2786 | 56.86 (45.11-74.09) | Mann-Whitney U test | <0.001 | <0.001 | 0.215 |
| Sex | Female | 481 | 206 (42.8%) | 2786 | 1119 (40.2%) | Chi-square test | 0.272 | 0.327 | 0.054 |
|  | Male |  | 275 (57.2%) |  | 1667 (59.8%) |  |  |  |  |
| Grade | Grade 1 | 323 | 88 (27.2%) | 2513 | 438 (17.4%) | Chi-square test | <0.001 | <0.001 | 0.305 |
|  | Grade 2 |  | 211 (65.3%) |  | 1981 (78.8%) |  |  |  |  |
|  | Grade 3 |  | 18 (5.6%) |  | 79 (3.1%) |  |  |  |  |
|  | Grade 4 |  | 6 (1.9%) |  | 15 (0.6%) |  |  |  |  |
| TNM stage | Stage I | 480 | 95 (19.8%) | 2771 | 439 (15.8%) | Chi-square test | <0.001 | <0.001 | 0.427 |
|  | Stage II |  | 139 (29.0%) |  | 1162 (41.9%) |  |  |  |  |
|  | Stage III |  | 147 (30.6%) |  | 994 (35.9%) |  |  |  |  |
|  | Stage IV |  | 99 (20.6%) |  | 176 (6.4%) |  |  |  |  |
| Tumor side | Left-sided | 481 | 365 (75.9%) | 2780 | 2138 (76.9%) | Chi-square test | 0.624 | 0.624 | 0.024 |
|  | Right-sided |  | 116 (24.1%) |  | 642 (23.1%) |  |  |  |  |
| MSI/MMR status | msi | 463 | 42 (9.1%) | 2598 | 200 (7.7%) | Chi-square test | 0.313 | 0.342 | 0.050 |
|  | mss |  | 421 (90.9%) |  | 2398 (92.3%) |  |  |  |  |
| CT phenotype | CT-S1 | 481 | 243 (50.5%) | 2786 | 1592 (57.1%) | Chi-square test | 0.007 | 0.010 | 0.133 |
|  | CT-S2 |  | 238 (49.5%) |  | 1194 (42.9%) |  |  |  |  |
| DFS event | Event | 481 | 185 (38.5%) | 2786 | 645 (23.2%) | Chi-square test | <0.001 | <0.001 | 0.336 |
|  | No event |  | 296 (61.5%) |  | 2141 (76.8%) |  |  |  |  |
| OS event | Event | 481 | 138 (28.7%) | 2786 | 374 (13.4%) | Chi-square test | <0.001 | <0.001 | 0.381 |
|  | No event |  | 343 (71.3%) |  | 2412 (86.6%) |  |  |  |  |

*Abbreviations: BH-FDR, Benjamini-Hochberg false discovery rate; BMI, body mass index; CMS, consensus molecular subtype; CT, computed tomography; DFS, disease-free survival; IQR, interquartile range; MMR, mismatch repair; MSI, microsatellite instability; MSS, microsatellite stable; OS, overall survival; SMD, standardized mean difference; SYSU6H, Sixth Affiliated Hospital of Sun Yat-sen University; TNM, tumor-node-metastasis.*

**Supplementary Table 6B. Baseline characteristics by Mutation data availability within SYSU6H cohort 2 (available n = 462; unavailable n = 2805)**

| **Variable** | **Level** | **Available non-missing N** | **Available, median (IQR) or n (%)** | **Unavailable non-missing N** | **Unavailable, median (IQR) or n (%)** | **Test** | **P value** | **BH-FDR** | **Absolute SMD** |
| --- | --- | --- | --- | --- | --- | --- | --- | --- | --- |
| Age, years |  | 340 | 58.00 (48.75-68.00) | 2732 | 60.00 (50.00-67.25) | Mann-Whitney U test | 0.152 | 0.196 | 0.085 |
| BMI, kg/m^2 |  | 458 | 22.49 (20.20-24.48) | 2760 | 22.72 (20.76-24.84) | Mann-Whitney U test | 0.01 | 0.017 | 0.160 |
| Tumor volume, cm^3 |  | 462 | 26.46 (14.29-50.27) | 2805 | 20.16 (9.59-40.72) | Mann-Whitney U test | <0.001 | <0.001 | 0.019 |
| Maximum axial diameter, mm |  | 462 | 50.98 (41.95-65.60) | 2805 | 56.86 (45.10-74.11) | Mann-Whitney U test | <0.001 | <0.001 | 0.234 |
| Sex | Female | 462 | 201 (43.5%) | 2805 | 1124 (40.1%) | Chi-square test | 0.163 | 0.196 | 0.070 |
|  | Male |  | 261 (56.5%) |  | 1681 (59.9%) |  |  |  |  |
| Grade | Grade 1 | 312 | 86 (27.6%) | 2524 | 440 (17.4%) | Chi-square test | <0.001 | <0.001 | 0.302 |
|  | Grade 2 |  | 204 (65.4%) |  | 1988 (78.8%) |  |  |  |  |
|  | Grade 3 |  | 17 (5.4%) |  | 80 (3.2%) |  |  |  |  |
|  | Grade 4 |  | 5 (1.6%) |  | 16 (0.6%) |  |  |  |  |
| TNM stage | Stage I | 461 | 94 (20.4%) | 2790 | 440 (15.8%) | Chi-square test | <0.001 | <0.001 | 0.369 |
|  | Stage II |  | 135 (29.3%) |  | 1166 (41.8%) |  |  |  |  |
|  | Stage III |  | 145 (31.5%) |  | 996 (35.7%) |  |  |  |  |
|  | Stage IV |  | 87 (18.9%) |  | 188 (6.7%) |  |  |  |  |
| Tumor side | Left-sided | 462 | 352 (76.2%) | 2799 | 2151 (76.8%) | Chi-square test | 0.756 | 0.756 | 0.016 |
|  | Right-sided |  | 110 (23.8%) |  | 648 (23.2%) |  |  |  |  |
| MSI/MMR status | msi | 445 | 40 (9.0%) | 2616 | 202 (7.7%) | Chi-square test | 0.36 | 0.393 | 0.046 |
|  | mss |  | 405 (91.0%) |  | 2414 (92.3%) |  |  |  |  |
| CT phenotype | CT-S1 | 462 | 236 (51.1%) | 2805 | 1599 (57.0%) | Chi-square test | 0.017 | 0.026 | 0.119 |
|  | CT-S2 |  | 226 (48.9%) |  | 1206 (43.0%) |  |  |  |  |
| DFS event | Event | 462 | 174 (37.7%) | 2805 | 656 (23.4%) | Chi-square test | <0.001 | <0.001 | 0.314 |
|  | No event |  | 288 (62.3%) |  | 2149 (76.6%) |  |  |  |  |
| OS event | Event | 462 | 132 (28.6%) | 2805 | 380 (13.5%) | Chi-square test | <0.001 | <0.001 | 0.375 |
|  | No event |  | 330 (71.4%) |  | 2425 (86.5%) |  |  |  |  |

*Abbreviations: BH-FDR, Benjamini-Hochberg false discovery rate; BMI, body mass index; CT, computed tomography; DFS, disease-free survival; IQR, interquartile range; MMR, mismatch repair; MSI, microsatellite instability; MSS, microsatellite stable; OS, overall survival; SMD, standardized mean difference; SYSU6H, Sixth Affiliated Hospital of Sun Yat-sen University; TNM, tumor-node-metastasis.*

**Supplementary Table 6C. Baseline characteristics by Ki-67 data availability within SYSU6H cohort 2 (available n = 272; unavailable n = 2995)**

| **Variable** | **Level** | **Available non-missing N** | **Available, median (IQR) or n (%)** | **Unavailable non-missing N** | **Unavailable, median (IQR) or n (%)** | **Test** | **P value** | **BH-FDR** | **Absolute SMD** |
| --- | --- | --- | --- | --- | --- | --- | --- | --- | --- |
| Age, years |  | 271 | 57.00 (48.00-67.00) | 2801 | 60.00 (50.00-68.00) | Mann-Whitney U test | 0.028 | 0.034 | 0.137 |
| BMI, kg/m^2 |  | 269 | 22.06 (19.83-23.88) | 2949 | 22.77 (20.76-24.91) | Mann-Whitney U test | <0.001 | <0.001 | 0.305 |
| Tumor volume, cm^3 |  | 272 | 31.38 (19.14-58.33) | 2995 | 20.14 (9.61-40.75) | Mann-Whitney U test | <0.001 | <0.001 | 0.107 |
| Maximum axial diameter, mm |  | 272 | 52.48 (43.54-65.70) | 2995 | 56.44 (44.64-73.41) | Mann-Whitney U test | 0.011 | 0.015 | 0.163 |
| Sex | Female | 272 | 117 (43.0%) | 2995 | 1208 (40.3%) | Chi-square test | 0.389 | 0.389 | 0.054 |
|  | Male |  | 155 (57.0%) |  | 1787 (59.7%) |  |  |  |  |
| Grade | Grade 1 | 246 | 72 (29.3%) | 2590 | 454 (17.5%) | Chi-square test | <0.001 | <0.001 | 0.340 |
|  | Grade 2 |  | 156 (63.4%) |  | 2036 (78.6%) |  |  |  |  |
|  | Grade 3 |  | 12 (4.9%) |  | 85 (3.3%) |  |  |  |  |
|  | Grade 4 |  | 6 (2.4%) |  | 15 (0.6%) |  |  |  |  |
| TNM stage | Stage I | 272 | 29 (10.7%) | 2979 | 505 (17.0%) | Chi-square test | <0.001 | <0.001 | 0.605 |
|  | Stage II |  | 89 (32.7%) |  | 1212 (40.7%) |  |  |  |  |
|  | Stage III |  | 76 (27.9%) |  | 1065 (35.8%) |  |  |  |  |
|  | Stage IV |  | 78 (28.7%) |  | 197 (6.6%) |  |  |  |  |
| Tumor side | Left-sided | 272 | 189 (69.5%) | 2989 | 2314 (77.4%) | Chi-square test | 0.003 | 0.005 | 0.180 |
|  | Right-sided |  | 83 (30.5%) |  | 675 (22.6%) |  |  |  |  |
| MSI/MMR status | msi | 262 | 25 (9.5%) | 2799 | 217 (7.8%) | Chi-square test | 0.305 | 0.332 | 0.064 |
|  | mss |  | 237 (90.5%) |  | 2582 (92.2%) |  |  |  |  |
| CT phenotype | CT-S1 | 272 | 129 (47.4%) | 2995 | 1706 (57.0%) | Chi-square test | 0.002 | 0.004 | 0.192 |
|  | CT-S2 |  | 143 (52.6%) |  | 1289 (43.0%) |  |  |  |  |
| DFS event | Event | 272 | 118 (43.4%) | 2995 | 712 (23.8%) | Chi-square test | <0.001 | <0.001 | 0.424 |
|  | No event |  | 154 (56.6%) |  | 2283 (76.2%) |  |  |  |  |
| OS event | Event | 272 | 89 (32.7%) | 2995 | 423 (14.1%) | Chi-square test | <0.001 | <0.001 | 0.450 |
|  | No event |  | 183 (67.3%) |  | 2572 (85.9%) |  |  |  |  |

*Note: Continuous variables are shown as median (IQR; interquartile range) and compared using two-sided Mann-Whitney U tests. Categorical variables are shown as n (%) and compared using chi-square or Fisher's exact tests as appropriate. BH-FDR values were calculated within each assay panel. Absolute standardized mean differences (SMDs) summarize the magnitude of imbalance; for categorical variables, the largest level-specific absolute SMD is shown on the first row of the variable. Abbreviations: BMI, body mass index; CT, computed tomography; DFS, disease-free survival; MMR, mismatch repair; MSI, microsatellite instability; MSS, microsatellite stable; OS, overall survival; SYSU6H, Sixth Affiliated Hospital of Sun Yat-sen University; TNM, tumor-node-metastasis.*

**Supplementary Table 7A. IPCW 3-year performance of clinical models**

| **Evaluation** | **Endpoint** | **Model** | **Horizon, years** | **N** | **Total events** | **Events by 3 years** | **IPCW Brier** | **IPCW AUC** | **G(3)** |
| --- | --- | --- | --- | --- | --- | --- | --- | --- | --- |
| Ningbo external | DFS | Clinical base | 3 | 400 | 72 | 70 | 0.143 | 0.805 | 0.318 |
| Ningbo external | DFS | Clinical base + CT phenotype | 3 | 400 | 72 | 70 | 0.105 | 0.902 | 0.318 |
| Ningbo external | OS | Clinical base | 3 | 404 | 42 | 37 | 0.099 | 0.758 | 0.348 |
| Ningbo external | OS | Clinical base + CT phenotype | 3 | 404 | 42 | 37 | 0.085 | 0.858 | 0.348 |
| SYSU6H 5-fold CV | DFS | Clinical base | 3 | 2657 | 620 | 501 | 0.130 | 0.731 | 0.753 |
| SYSU6H 5-fold CV | DFS | Clinical base + CT phenotype | 3 | 2657 | 620 | 501 | 0.108 | 0.855 | 0.753 |
| SYSU6H 5-fold CV | OS | Clinical base | 3 | 2657 | 348 | 219 | 0.067 | 0.793 | 0.754 |
| SYSU6H 5-fold CV | OS | Clinical base + CT phenotype | 3 | 2657 | 348 | 219 | 0.059 | 0.894 | 0.754 |

*Abbreviations: AUC, area under the receiver operating characteristic curve; CT, computed tomography; CV, cross-validation; DFS, disease-free survival; IPCW, inverse probability of censoring weighting; OS, overall survival; SYSU6H, Sixth Affiliated Hospital of Sun Yat-sen University. G(3), estimated probability of remaining uncensored at 3 years.*

**Supplementary Table 7B. Incremental performance and IPCW continuous NRI/IDI after adding CT phenotype**

| **Evaluation** | **Endpoint** | **Horizon, years** | **New model** | **Reference model** | **Delta AUC** | **Delta Brier** | **Continuous NRI** | **NRI events** | **NRI non-events** | **IDI** |
| --- | --- | --- | --- | --- | --- | --- | --- | --- | --- | --- |
| Ningbo external | DFS | 3 | Clinical base + CT phenotype | Clinical base | 0.097 | -0.038 | 1.136 | 0.762 | 0.374 | 0.154 |
| Ningbo external | OS | 3 | Clinical base + CT phenotype | Clinical base | 0.101 | -0.013 | 0.794 | 0.575 | 0.220 | 0.054 |
| SYSU6H 5-fold CV | DFS | 3 | Clinical base + CT phenotype | Clinical base | 0.124 | -0.021 | 1.152 | 0.777 | 0.375 | 0.133 |
| SYSU6H 5-fold CV | OS | 3 | Clinical base + CT phenotype | Clinical base | 0.101 | -0.007 | 1.162 | 0.900 | 0.262 | 0.079 |

*Note: The clinical base Cox model included age, sex, tumor grade, TNM stage, tumor side, and MSI status. The augmented model added the locked CT phenotype. Analyses used complete cases and a fixed 3-year horizon. SYSU6H estimates conditionally cross-validated the Cox layer using fixed phenotype labels, with training-fold-only age standardization, model fitting, and baseline-risk estimation; Ningbo estimates were generated by models fitted in SYSU6H cohort 2 and evaluated without refitting. IPCW censoring distributions were estimated by Kaplan-Meier analysis without weight truncation; G(3) is the estimated probability of remaining uncensored at 3 years. Calibration used deciles of predicted risk, and decision-curve analysis used thresholds from 0.05 to 0.50. Delta AUC and delta Brier are augmented minus clinical base; a negative delta Brier indicates lower prediction error. Continuous NRI and IDI are IPCW point estimates. IPCW, inverse probability of censoring weighting; NRI, net reclassification improvement; IDI, integrated discrimination improvement. Internal evaluation did not repeat phenotype derivation within folds and is not an end-to-end validation of phenotype derivation. Abbreviations: AUC, area under the receiver operating characteristic curve; CT, computed tomography; CV, cross-validation; DFS, disease-free survival; MSI, microsatellite instability; OS, overall survival; SYSU6H, Sixth Affiliated Hospital of Sun Yat-sen University; TNM, tumor-node-metastasis.*
